# Scar‐Degrading Endothelial Cells as a Treatment for Advanced Liver Fibrosis

**DOI:** 10.1002/advs.202203315

**Published:** 2022-12-09

**Authors:** Peng Zhao, Tian Sun, Cheng Lyu, Kaini Liang, Yudi Niu, Yuying Zhang, Chenhui Cao, Canhong Xiang, Yanan Du

**Affiliations:** ^1^ Department of Biomedical Engineering School of Medicine Tsinghua‐Peking Center for Life Sciences Tsinghua University Beijing 100084 China; ^2^ Department of Hepatobiliary Surgery Beijing Tsinghua Changgung Hospital Beijing 100084 China

**Keywords:** extracellular matrix degradation, high‐throughput screening system, liver fibrosis, liver sinusoidal endothelial cells

## Abstract

Deposition of extracellular matrix (ECM) in the liver is an important feature of liver cirrhosis. Recovery from liver cirrhosis is physiologically challenging, partially due to the ECM in scar tissue showing resistance to cell‐mediated degradation by secreted matrix metalloproteinases (MMPs). Here, a cell‐mediated ECM‐degradation screening system (CEDSS) in vitro is constructed for high‐throughput searching for cells with tremendous degradation ability. ECM‐degrading liver sinusoidal endothelial cells (dLSECs) are screened using CEDSS, which exhibit 17 times the ability to degrade collagen when compared to other cells. The degradation ability of dLSECs is mediated by the upregulation of MMP9. In particular, mRNA expression of MMP9 shows an 833‐fold increase in dLSECs compared to normal endothelial cells (nLSECs), and MMP9 is regulated by transcription factor c‐Fos. In vivo, single intrasplenic injection of dLSECs alleviates advanced liver fibrosis in mice, while intraperitoneal administration of liver‐targeting peptide‐modified dLSECs shows enhanced fibrosis‐targeting effects. Degradative human umbilical vein endothelial cells (dHUVECs) prove their enhanced potential of clinical translation. Together, these results highlight the potential of ECM‐degrading endothelial cells in alleviating advanced liver fibrosis, thus providing remarkable insights in the development of ECM‐targeting therapeutics.

## Graphical abstract

Extracellular matrix (ECM) scar hinders the recovery from liver cirrhosis. Here, an in vitro cell‐mediated ECM‐degradation screening system (CEDSS) is developed and discovers a type of primed endothelial cells with exceptional ECM‐degradation ability (dLSECs) after high‐throughput screening. dLSECs can effectively breakdown collagen scar in vitro and in vivo through matrix metalloproteinase (MMP) expression, providing potential ECM‐targeting strategy for cirrhosis treatment.

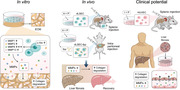

## 1 Introduction

Chronic liver injury could lead to advanced liver fibrosis and eventually liver cirrhosis, which is a major deadly disease worldwide currently lacking in effective clinical treatments.^[^ ^1^ ^]^ As liver fibrosis progresses, extracellular matrix (ECM), in particular collagen type I, is heavily deposited into hepatic tissue, which is caused by the imbalance between collagen synthesis and degradation.^[^ ^2^, ^3^, ^4^ ^]^ Recovery from liver cirrhosis is challenging because scar tissue is resistant to cell‐mediated degradation, underscoring the significance of developing new treatment strategies that can target this aberrant collagen property.^[^ ^5^ ^]^

MMPs are largely responsible for degrading deposited collagen and contributing to ECM hemostasis together with inhibitors of MMP activity known as tissue inhibitor of metalloproteinases (TIMPs).^[^ ^6^ ^]^ Excessive collagen accumulation can occur due to an imbalance between MMPs and TIMPs.^[^ ^7^ ^]^ Dysregulation of the balance between MMPs and TIMPs has been shown to play an important role in the progression of liver fibrosis.^[^ ^8^, ^9^ ^]^

MMPs can be secreted by various types of cells, such as endothelial cells, macrophages, fibroblasts, and neutrophils. Specifically, human umbilical vein endothelial cells (HUVECs) secreted active tissue proteinase B, MMP2, and MMP14 to degrade ECM during vascular tube formation.^[^ ^10^ ^]^ During sprouting angiogenesis, endothelial cells secreted MMP2 and MMP9 to degrade the basement membrane, allowing them to invade local tissues and promote further endothelial cell migration.^[^ ^11^ ^]^ Differing from soluble MMPs, membrane type‐1 matrix metalloproteinase (MMP14) was a membrane‐bound MMP that could be activated by growth factors and chemokines and played an important role in driving neovessel formation.^[^ ^12^ ^]^ M2‐like macrophages, along with Col1a1‐expressing fibroblasts, have been shown to be crucial in the uptake of collagen in vivo.^[^ ^13^ ^]^ During proteolytic degradation of ECM by macrophages, MMP14 localized to podosomes played a critical role in the matrix degradation process.^[^ ^14^ ^]^ In a liver fibrosis mouse model, CD11b^hi^F4/80^int^Ly6C^lo^ macrophages were shown to be a dominant force in the resolution of liver fibrosis through their expression of MMP9 and MMP12.^[^ ^15^ ^]^ Macrophages underwent phenotypic conversion when cocultured with mesenchymal stromal cells (MSCs) and administration of macrophages together with MSCs promoted MMP expression in a fibrotic liver, which led to alleviation of mouse liver fibrosis.^[^ ^16^ ^]^ In dense collagen hydrogel, MMPs secreted by fibroblasts have been shown to degrade the local ECM to promote the migration of macrophages.^[^ ^17^ ^]^ In response to an inflammatory environment, MSCs secreted several isoforms of MMPs that resulted in degradation of the ECM.^[^ ^18^ ^]^ Moreover, exosomes served as vehicles for the transport of MMPs and elastase‐containing exosomes secreted by neutrophils have been shown to degrade local ECM in lung.^[^ ^19^ ^]^ Up until now, no evidence has been reported on the effectiveness of ECM‐degradation therapy and a challenge in studying this has been the lack of methods for empowering cells with an enhanced ability to degrade ECM in vitro.

**Figure 1:**
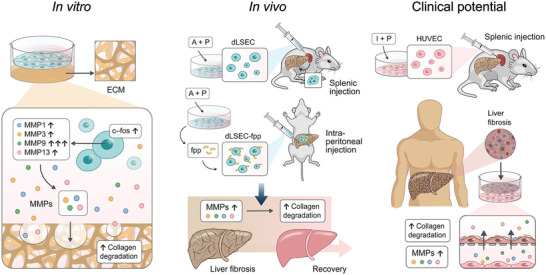
Schematic of screening highly ECM‐degrading cells, dLSECs, by developing CEDSS and validation of ECM degradation ability of dLSECs in vitro and in vivo, which shows potential for liver cirrhosis treatment.

In this work, we constructed a high‐throughput platform to precisely quantify the collagen degradation ability of cells (CEDSS). We then screened these cells to identify potential cell types and degradation priming conditions to increase cells’ ability to breakdown ECM in vitro. We successfully screened out and obtained ECM‐degrading LSECs (dLSECs) using CEDSS, which showed an ≈17‐fold increase in collagen degradation ability compared to other candidate cell types. The ECM degradation ability of dLSECs was due to an upregulation in the expression of MMPs, especially MMP9, which was regulated by transcription factor c‐Fos. We verified that dLSECs showed great potential for treating advanced liver fibrosis using two animal models. Similar approaches were used to construct dHUVECs and dHUVEC treatment could also effectively alleviate advanced liver fibrosis. Moreover, collagen scar tissue in clinical liver cirrhosis samples could be degraded by dHUVEC treatment ex vivo. Together, our results provide a robust cell therapy strategy based on ECM degradation facilitated by cells, which can be used in the treatment of liver cirrhosis (**Figure** **1**).

## 2 Results

### 2.1 Liver Sinusoidal Endothelial Cells have a Notable Ability to Degrade Collagen Following Stimulation by Accutase and Phorbol Ester (PMA)

**Figure 2:**
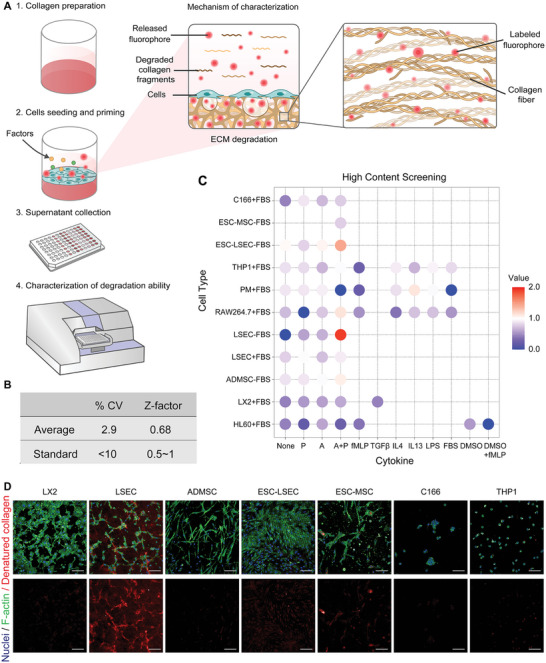
Construction of CEDSS and screening of dLSECs with highly ECM degradation ability. A) Schematic of screening cells with highly ECM degradation ability by using CEDSS. Details were shown in Methods part. B) CV and Z‐factor of CEDSS. C) Collagen degradation ability of cell candidates characterized by CEDSS. D) Representative high‐content fluorescent images showing the cell‐mediated collagen degradation stained by CHP assay (stimulating by accutase and PMA). Nuclei (blue), F‐actin (green), denatured collagen (red). Scar bars, 100 µm.

To identify satisfactory cell candidates with significant ECM degradation ability, we first constructed an engineered CEDSS for high‐throughput detection of cellular degradation ability in vitro. Type I collagen, which is the dominant type of ECM present in fibrotic disease,^[^ ^2^ ^]^ was conjugated to NHS‐Rhodamine and used to fabricate collagen matrix substrate. Cell candidates were seeded onto the collagen matrix and cell‐matrix interactions, such as collagen degradation, were allowed to occur. For most conditions without cell‐mediated degradation, the collagen matrix substrate was kept intact and Rhodamine molecules were still bound to the collagen substrate resulting in minimum fluorescent signals detected in the culture medium. Once a cell candidate could degrade the collagen matrix, the collagen structure was broken down into fragments, releasing the conjugated fluorescent rhodamine molecules into the culture medium. Therefore, the increase of fluorescent signals in the cell culture medium showed a high linear correlation with the degradation proportion of collagen by cell candidates. The rhodamine‐conjugated collagen matrix could be custom‐prepared in the commercialized 96‐well or 384‐well plates, which made it a simple and practical method for high‐throughput detection by multi‐well spectrophotometer (**Figure** **2**A). To investigate the quality and robustness of CEDSS, Z‐factor was calculated. Uniformity was represented by the coefficient of variance (CV) of the fluorescent signals produced from absolutely degraded collagen matrices. The stability of the CEDSS was verified by CV value of 0.029 and Z‐factor of 0.68 (Figure 2B). In high‐throughput screening, CV less than 10% and Z‐factor between 0.5 and 1 represented the minimal variance, which indicated the good performance of the system. Based on CEDSS, we screened 60 cell populations with potential function of ECM degradation combined with different stimulating strategies, and identified a candidate showing surprisingly high ECM degradation ability with approximately 17‐fold increase in collagen degradation ability compared to other candidates, this population was labeled as dLSECs (Figure 2C). The potent ECM degradation ability of dLSECs was induced by treating liver sinusoidal endothelial cells with a combination of stimulating factors, including accutase (A) and PMA (P). A and P were chosen in the screening library since they could play an important role in regulating ECM (e.g., collagen) remodeling, in particular leading to the increased MMP expression.^[^ ^20^, ^21^, ^22^ ^]^ And stimulation by vehicles was used to obtain normal liver sinusoidal endothelial cells (nLSECs). In addition, the part of collagen being degraded could be stained by Collagen Hybridizing Peptide (CHP) assay and visualized by a high‐content imaging system (Figure 2D; Figure S1 and Table S1, Supporting Information). Consistent with results of CEDSS, dLSECs showed the highest ECM degradation ability indicated by the highest CHP signals compared with other candidates.

### 2.2 dLSECs Demonstrate a Powerful Ability to Degrade Collagen

**Figure 3:**
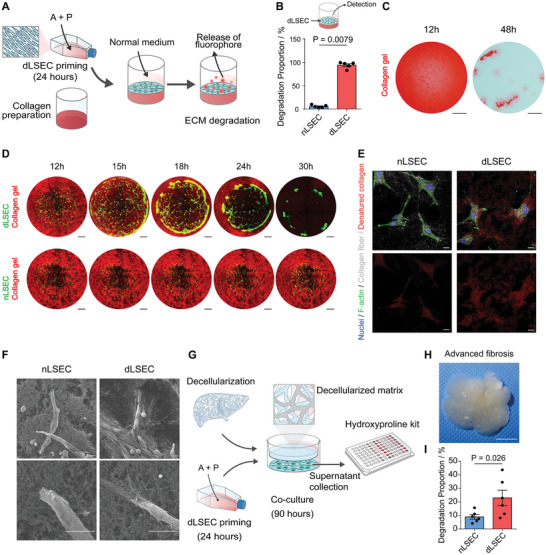
dLSECs showed highly ECM degradation ability in vitro. A) Schematic of constructing dLSECs and characterizing the ECM degradation ability by CEDSS. B) Statistical analysis of collagen degradation mediated by nLSECs and dLSECs (*n* = 5, biological independent samples). C) Ponceau S staining of collagen matrix after 12 and 48 h’ degradation mediated by dLSECs. Scale bars, 100 µm. D) Representative images of dynamic degradation of collagen matrix mediated by nLSEC‐mVenus‐Aka and dLSEC‐mVenus‐Aka. dLSEC‐mVenus‐Aka, nLSEC‐mVenus‐Aka (green). Collagen I (red). Scale bars, 500 µm. See also Movies S1 and S2 (Supporting Information). E) Representative fluorescent images of CHP staining of collagen matrix with degradation mediated by nLSECs and dLSECs. Top panels, F‐actin (green), nuclei (blue), collagen fibrils (white); bottom panels, denatured collagen (red). Scale bars, 20 µm. F) Representative SEM images of collagen matrix with degradation mediated by nLSECs and dLSECs. Scale bars, 10 µm. G) Schematic of degrading the mouse‐whole‐liver‐decellularized ECM by dLSECs and nLSECs. H) Representative bright‐field images of liver decellularized ECM obtained from mouse with advanced liver fibrosis. Scale bars, 1 cm. I) Statistical analysis of the degradation of advanced‐fibrotic liver decellularized ECM mediated by nLSECs and dLSECs. (*n* = 6, biological independent samples). Statistical analysis was performed using two‐tailed unpaired *t*‐test. Results are presented as means ± SEM.

To test the ability of dLSECs to continually make MMPs and degrade collagen, which is essential for long‐term effect in vivo, we characterized the longevity of ECM degradation by dLSECs by removing A and P factors after 24 h of priming (**Figure** **3**A). We found that dLSECs showed extended morphologies, with higher aspect ratio and cell area and lower roundness compared with nLSECs (Figure S2A,B, Supporting Information). Quantitative analysis of ECM degradation ability showed that dLSECs could degrade 80% of the collagen matrix in 48 h, which was 12‐fold more than that of nLSECs (Figure 3B; Figure S3, Supporting Information). The degradation of collagen matrices mediated by dLSECs could also be observed at a macroscopic level, reflected in the degradation of the entire collagen matrix in 48 h by dLSECs (whereas the collagen matrix maintained the intact structure in nLSEC group) (Figure 3C). Next, we characterized the dynamic ECM degradation process by live imaging. The collagen matrix in the peripheral area was gradually degraded by dLSECs and dLSECs were contracted together followed by being released into the cultured medium due to a total degradation of the collagen matrix. In contrast, the condition of the collagen matrix and nLSECs was unchanged during the same period of time (Figure 3D; Movies S1 and S2, Supporting Information). Consistently, a strong CHP signal could be observed in the area surrounding the dLSECs, but not in the area surrounding the nLSECs (Figure 3E; Figure S4, Supporting Information). Scanning electron microscopy (SEM) imaging showed a number of holes could be observed adjacent to the protrusions, which indicated that the collagen matrix had been degraded. In contrast, collagen matrices showed dense structure adjacent to nLSECs (Figure 3F). What's more, SEM and transmission electron microscopy (TEM) imaging showed that the protrusions of dLSECs could bind collagen fibrils and the strong cell‐ECM interactions were also verified by high expression of paxillin in protrusion of dLSECs, which was not observed in nLSECs (Figure 3F; Figure S5, Supporting Information). What's more, the ability of tube formation of dLSECs was similar to nLSECs (Figure S6, Supporting Information). To further confirm the ability of dLSECs to degrade the fibrotic ECM in seen in cirrhotic livers, we isolated whole liver decellularized ECM from mice with advanced liver fibrosis induced by CCl_4_, and then characterized whether dLSECs could degrade fibrotic liver ECM ex vivo (Figure 3G). The advanced fibrotic liver ECM showed dense inner structure (Figure 3H). By priming the liver ECM with cultured nLSECs and dLSECs, we found that 23% of advanced fibrotic liver ECM could be degraded by dLSECs in 90 h, which was 2.6 times more than that degraded by nLSECs (Figure 3I). These results collectively demonstrate the potent ECM degradation ability of dLSECs both in vitro and ex vivo.

### 2.3 MMPs Secreted by dLSECs Play an Important Role in Degrading Collagen

**Figure 4:**
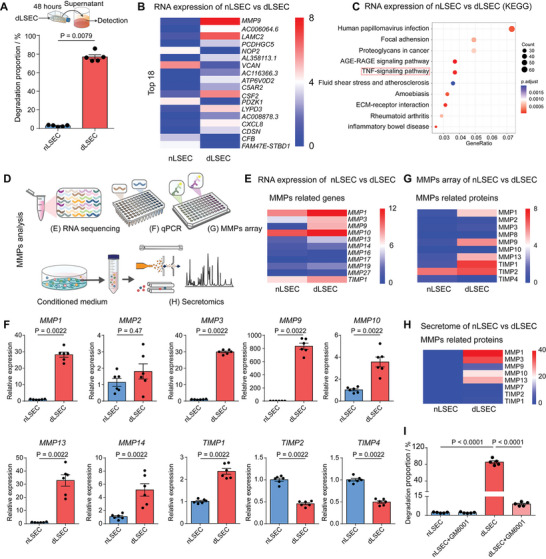
dLSECs showed high expression of MMPs. A) Characterization of ECM degradation ability of supernatant of nLSECs and dLSECs in normal medium for 48 h (*n* = 5, biological independent samples). B) Heatmap view of top 18 differentially expressed genes in dLSECs compared with nLSECs based on logarithmic transformation of FPKM counts which is log_10_ (FPKM+1). The relative abundance of gene expression was indicated by transition from blue (the lowest), white (middle), and red (the highest). Analyzed by RNA‐seq assays. C) KEGG pathway analysis of differentially expressed genes in dLSECs compared with nLSECs. Analyzed by RNA‐seq assays. D) Schematic of characterizing the expression and function of MMPs in dLSECs. E) Heatmap view of differentially expressed genes involved in MMPs based on logarithmic transformation of FPKM counts which is log_10_ (FPKM +1). The relative abundance of gene expression was indicated by transition from blue (the lowest), white (middle), and red (the highest). Analyzed by RNA‐seq assays. F) Relative mRNA expression of *MMP1, MMP2, MMP3, MMP9, MMP10, MMP13, MMP14, TIMP1, TIMP2*, and *TIMP4*. Analyzed by qPCR assay (*n* = 6, biological independent samples). G) Heatmap view of secreted MMPs‐related proteins in supernatants of dLSECs and nLSECs based on logarithmic transformation of protein counts which is log_10_ (FPKM+1). The relative abundance of protein expression was indicated by transition from blue (the lowest), white (middle) and red (the highest). Analyzed by MMPs protein array. H) Heatmap view of secreted MMPs‐related proteins in supernatants of dLSECs and nLSECs based on logarithmic transformation of secretome counts which is log_10_ (FPKM+1). The relative abundance of gene expression was indicated by transition from blue (the lowest), white (middle), and red (the highest). Analyzed by secretomics. I) Characterization of ECM degradation ability of dLSECs with or without GM6001 (*n* = 5, biological independent samples). The statistical analysis in (A,F) was performed using two‐tailed unpaired *t*‐test. The statistical analysis in (I) was performed using a one‐way ANOVA with Turkey test. Results are presented as means ± SEM.

We next sought to determine by which mechanism the dLSECs mediated degradation of the collagen matrix. We collected the conditioned medium from dLSECs and nLSECs and characterized the collagen matrix degradation ability. Results showed that the conditioned medium from dLSECs led to 77% of collagen matrix degradation in 48 h, 29 times to that of nLSEC group, which indicated that secretions of dLSECs were responsible for the collagen matrix degradation (**Figure** **4**A). We then characterized the differential expression of genes in dLSECs and nLSECs by RNA sequencing (RNA‐seq) assay and found 255 differentially upregulated genes and 354 differentially downregulated genes (Figure S7A, Supporting Information). Gene ontology (GO) enrichment analysis indicated the differential genes were related to the structure and assembly of ECM (Figure S7B, Supporting Information). Surprisingly, we found that MMP9 was the most upregulated gene expressed by dLSECs compared with nLSECs (Figure 4B). Moreover, differential expression genes in dLSECs compared with nLSECs enriched in the TNF signaling pathway according to Kyoto Encyclopedia of Genes and Genomes (KEGG) analysis (Figure 4C). We then sought to determine whether dLSECs led to the ECM degradation by secreting MMPs (Figure 4D). By re‐analyzing the RNA‐seq results, we found that most MMP family genes were upregulated in dLSECs, which was further validated by quantitative polymerase chain reaction (qPCR) assay (Figure 4, E and F). Specifically, *MMP1, 3, 9, 10, 13, 14*, *and TIMP1* were all upregulated in dLSECs accompanied by downregulation of *TIMP2 and TIMP4*. In particular, mRNA expression of *MMP9* showed an 833‐fold increase in dLSECs compared to nLSECs (Figure 4F). The upregulation of MMP secretion in dLSECs was further confirmed by MMP protein array assay, which indicated the significant increase in expression of MMP1, 3, 9, and 13 (Figure 4G). These results were also validated by secretomics analysis (Figure 4H). What's more, COLIV and COL1A1 were downregulated accompanied by the upregulated FN1 according to secretomes analysis and immunofluorescent staining (Figure S8A–D, Supporting Information). Broad inhibitor of MMP secretion, GM6001, was used to treat dLSECs, which resulted in a significant decrease in the ECM degradation ability of dLSECs to a level comparable to that of nLSECs (Figure 4I). Taken together, these results suggest that secreted MMPs played a crucial role in the ECM degradation ability of dLSECs.

### 2.4 c‐Fos Regulates Expression of MMP9 in dLSECs

**Figure 5:**
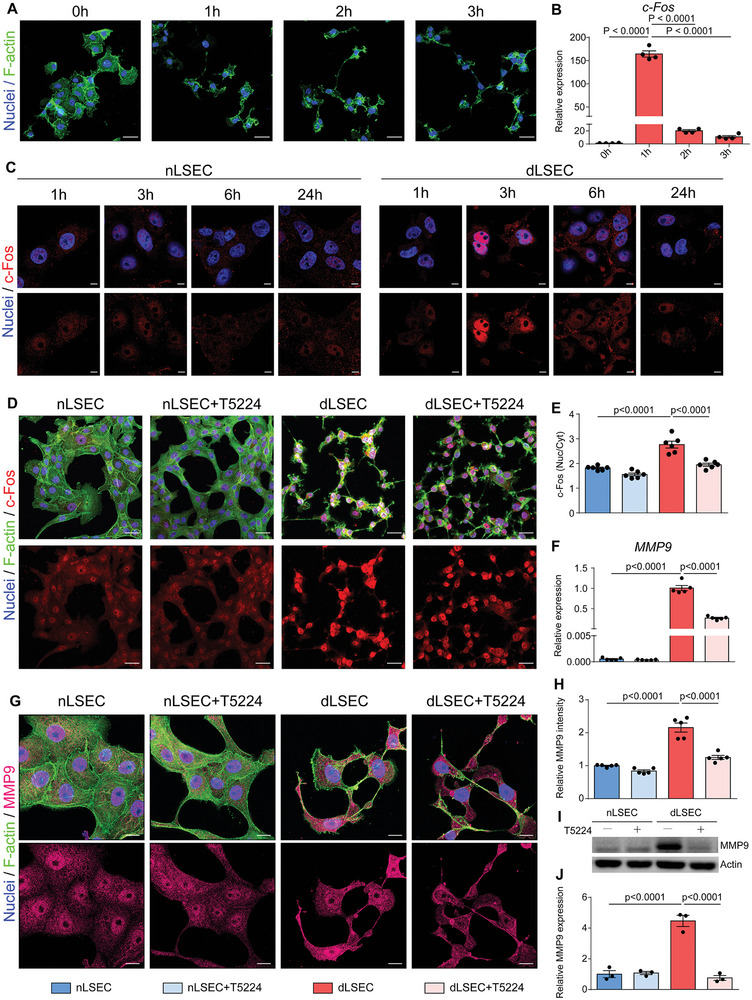
The highly expressed MMP9 in dLSECs was regulated by c‐Fos. A) Representative F‐actin staining of dLSECs in the following 3 h after stimulation. Nuclei (blue); F‐actin (green). Scale bars, 40 µm. B) Relative mRNA expression of c‐Fos in dLSECs in the following 3 h after stimulation by accutase and PMA (*n* = 4, biological independent samples). C) Representative images of c‐Fos staining of nLSECs and dLSECs during the 24 h stimulation. Nuclei (blue); c‐Fos (red). Scale bars, 10 µm. D) Representative images of c‐Fos staining of nLSECs and dLSECs with treatment of T5224. Top panel, nuclei (blue), F‐actin (green), c‐Fos (red). Bottom panels, c‐Fos (red). Scale bars, 40 µm. E) Statistical analysis of nuclear/cytoplasm ratio in (D) (n = 6, number of cells analyzed per condition). F) Relative mRNA expression of MMP9 of nLSECs and dLSECs with or without treatment of T5224 (*n* = 5, biological independent samples). G) Representative images of MMP9 staining in nLSECs and dLSECs with or without treatment of T5224. Top panel, nuclei (blue), F‐actin (green), MMP9 (magenta). Bottom panels, MMP9 (magenta). Scale bars, 20 µm. H) Statistical analysis of total MMP9 level in (G) (*n* = 5, number of cells analyzed per condition). I) Representative images of western blot of MMP9 expression in nLSECs and dLSECs with treatment of T5224. J) Statistical analysis of MMP9 from immunoblots as shown in (I) (*n* = 3, biological independent samples). The statistical analysis was performed using a one‐way ANOVA with Turkey test. Results are presented as means ± SEM.

Considering that MMP9 was the most upregulated gene in dLSECs at the RNA and protein level, we next sought to determine which factor was responsible for regulating MMP9 expression. By using KEGG analysis of the differentially expressed genes from our RNA‐seq data, we found that the transcription factor c‐Fos acts as a critical regulator of the TNF signaling pathway, of which multiple pathway members were found to be differentially expressed (Figure 4C; Figure S9, Supporting Information). Moreover, relative expression of representative genes regulated by c‐Fos were highly upregulated in dLSECs compared with nLSECs (Figure S10, Supporting Information). Therefore, we hypothesized that high expression of MMP9 was regulated by c‐Fos in dLSECs. We found that the morphology of dLSECs changed gradually in the 3 h after combined stimulation with A and P (A+P stimulation) (**Figure** **5**A). Gene expression of *c‐Fos* increased dramatically at 1 h and then showed sharp decrease in the following 2 h (Figure 5B). Immunofluorescent images showed that c‐Fos in dLSECs had significant nuclear localization by 3 h after A+P stimulation. By contrast, the expression and localization of c‐Fos in nLSECs did not change during the same period of time (Figure 5C; Figure S11, Supporting Information). This differential expression of c‐Fos in dLSECs was inhibited by treating dLSECs with a c‐Fos inhibitor, T5224, which decreased the expression of c‐Fos and MMP9 in dLSECs to the level similar to nLSECs (Figure 5D–J and Figure S12). In addition, the ECM‐degradation ability of dLSECs was also dramatically reduced after c‐Fos inhibition by using T5224 (Figure S13, Supporting Information). The mRNA expression of *MMP3, 13* in dLSECs was also downregulated by c‐Fos inhibition (Figure S14, Supporting Information). These results demonstrate that c‐Fos is upregulated and localizes to cell nucleus in dLSECs, which is responsible for upregulation of MMP9 expression during ECM degradation by dLSECs.

### 2.5 dLSECs Alleviate the Process of Advanced Liver Fibrosis

**Figure 6:**
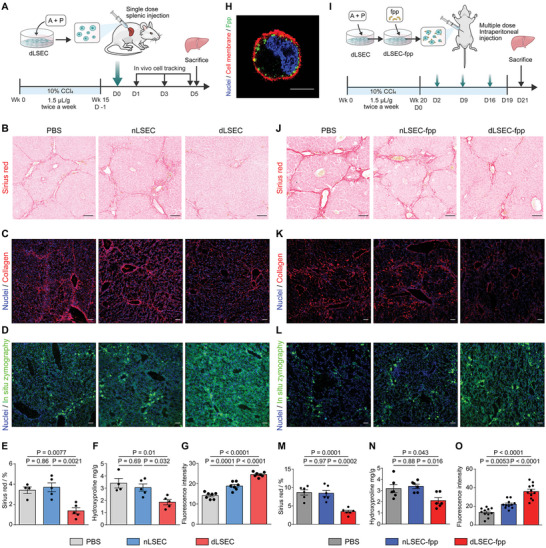
One‐dose intrasplenic administration of dLSECs or systemic administration of liver‐targeting dLSECs promoted the recovery from advanced liver fibrosis in mice. A) Schematic of one‐dose intrasplenic administration of dLSECs to treat the mice with advanced liver fibrosis induced by CCl_4_. B and E) Representative Sirius Red staining images and statistical analysis of collagen deposition in liver tissues treated by PBS, nLSECs and dLSECs (*n* ≥ 4, biological independent mice per group). Scale bars, 100 µm. C) Representative immunofluorescent images of collagen staining in livers tissues treated by PBS, nLSECs, and dLSECs. Scale bars, 100 µm. F) Quantification of hepatic hydroxyproline content. The data are expressed as hydroxyproline (mg) per liver dry weight (g) (*n* ≥ 4, biologically independent mice per group). D and G) Representative images and statistical analysis of MMPs stained by in situ zymography in liver tissues treated by PBS, nLSECs and dLSECs (*n* = 7, points of detection derived from at least 3 biologically independent mice). Scale bars, 100 µm. H) Representative fluorescent images of dLSEC‐fpp. Nuclei (blue), fpp (green), cell membrane (red). Scale bars, 10 µm. I) Schematic of systemic administration of dLSECs in treatment of progressive advanced liver fibrosis induced by CCl_4_. J and M) Representative Sirius Red staining images and statistical analysis of collagen deposition in liver tissues treated by PBS, nLSEC‐fpp and dLSEC‐fpp (*n* = 6, biological independent mice per group). Scale bars, 100 µm. K) Representative immunofluorescent images of collagen staining in liver tissues treated by PBS, nLSEC‐fpp, and dLSEC‐fpp. Scale bars, 100 µm. N) Quantification of hepatic hydroxyproline content. The data are expressed as hydroxyproline (mg) per liver dry weight (g) (*n* = 6, biologically independent mice per group). L and O) Representative images and statistical analysis of MMPs stained by in situ zymography in liver tissues treated by PBS, nLSEC‐fpp, and dLSEC‐fpp (*n* = 11, points of detection derived from at least 3 biologically independent mice). Scale bars, 100 µm. The statistical analysis was performed using a one‐way ANOVA with Turkey test. Results are presented as means ± SEM.

Considering that dLSECs could effectively degrade collagen matrix in vitro and ex vivo, we next sought to determine whether the ECM degradation effect would also occur in vivo and treat advanced liver fibrosis. We established an advanced liver fibrosis murine model by CCl_4_ induction twice a week for 15 weeks, and then treated the fibrotic liver by a one‐dose, intrasplenic injection of dLSECs (**Figure** **6**A). A number of injected dLSECs could be tracked to the liver at day 1 after injection, and the number of dLSECs decreased in the following days. We evaluated the anti‐fibrotic effect of this treatment on the 5th day post injection and found that a proportion of dLSECs still remained in the livers. Not only dLSECs, but also nLSECs remained in the liver until 5 days and the quantified bioluminescence is equal for both groups in all days tested (Figure S15, Supporting Information). By Sirius Red staining and immunofluorescent staining of collagen, we found a decrease in the number of collagen septa areas found in liver tissue treated by dLSECs compared with that treated by nLSECs and vehicle controls (Figure 6B,C,E). Consistently, a decreased level of hepatic hyodroxyproline in dLSECs‐treated livers also indicated the low level of collagen scar tissue deposition (Figure 6F). This treatment did not induce structural damage to other organs besides liver (Figure S16, Supporting Information). Moreover, results of in situ zymography assay showed that liver tissue treated with dLSECs had an increase in the expression of ECM‐degrading proteases compared to nLSECs and vehicle groups, indicating that dLSECs promoted the recovery from advanced liver fibrosis through the ECM degradation effect (Figure 6D,G). To verify the potential cross talk between dLSECs and hepatic stellate cells (HSCs) during the fibrosis treatment, we performed a conditioned medium assay by using human hepatic stellate cell line LX2. Results showed that LX‐2 treated by conditioned medium from dLSECs (dLX2s) exhibited a decreased expression of *α*‐smooth muscle actin (*α*SMA) and a decreased contraction of collagen compared with that treated by conditioned medium from nLSECs (nLX2s), accompanied by decreased expression levels of genes related to liver fibrosis progression, such as *ACTA2, COL1A1*, *and COL3A1* (Figure S17, Supporting Information). In addition, the expression of these pro‐fibrotic genes was also decreased in liver tissue treated by dLSECs compared with that treated by nLSECs and vehicle controls (Figure S18A–C, Supporting Information). Decrease in *α*SMA positive cells was also observed in liver tissue treated by dLSECs (Figure S18D, Supporting Information). What's more, dLSEC treatment did not induce an unexpected inflammatory response or angiogenesis in liver tissues. And no significant liver regeneration was observed after dLSEC treatment (Figures S19–23, Supporting Information). Taken together, these results suggest that one‐dose administration of dLSECs showed potential to promote the recovery from advanced liver fibrosis.

### 2.6 dLSECs Modified with Functional Peptide(dLSEC‐fpp) Target the Damaged Liver and show a Therapeutic Effect on Advanced Liver Fibrosis

Cirrhotic patients could benefit more from long‐term treatments compared with one‐dose treatments since the resolution of collagen scar tissue in the liver requires a period of time. To systemically administer the dLSEC to liver tissue, we engineered the dLSEC to improve the liver‐targeting abilities (Figure S24, Supporting Information). Through membrane modification of fpp, a liver‐targeting peptide, we have previously promoted MSCs to target to and stay in livers in a liver injury model.^[^ ^23^ ^]^ Likewise, we modified fpp to the membrane of dLSECs (dLSEC‐fpp) and nLSECs (nLSEC‐fpp), and found that dLSECs could be effectively modified by incubating cells with 1 mm fpp for 30 min (Figure 6H; Figure S25, Supporting Information). dLSEC‐fpp showed high ECM degradation ability comparable to the unmodified dLSECs (Figure S26, Supporting Information). We next validated the liver‐targeting characteristics of dLSEC‐fpp in a liver injury model through intraperitoneal administration of dLSEC‐fpp (Figure S24, Supporting Information). Anatomical imaging on the third post injection showed a significant increase in dLSEC‐fpp signals in the liver compared with unmodified dLSECs, indicating the improved liver‐targeting ability of dLSEC‐fpp (Figure S27, Supporting Information). To determine the potential of systemic administration of dLSEC‐fpp to alleviate progressive liver fibrosis, we established advanced liver fibrosis models by 20 weeks’ induction of CCl_4_ and the induction was continued to keep the progression of liver fibrosis in the following 3 weeks of dLSEC treatment. We then treated the ongoing advanced liver fibrosis by systemic administration of dLSEC‐fpp through intraperitoneal injection once a week and continued for 3 weeks along with CCl_4_ induction (Figure 6I; Figure S24, Supporting Information). A number of dLSEC‐fpp could be detected in livers on the 21st day post injection (Figure S28, Supporting Information). Both Sirius Red staining and immunofluorescent staining of collagen I showed the decreased deposited collagen‐rich scar tissue in the liver treated by dLSEC‐fpp compared with that in liver treated by nLSEC‐fpp and vehicle controls (Figure 6J, K,M). Consistently, the expression of hepatic hydroxyproline was the lowest in dLSEC‐fpp‐treated group (Figure 6N). Results of in situ zymography assay also indicated an elevated expression of ECM‐degrading proteases in liver tissue treated by dLSEC‐fpp (Figure 6L,O). The expression of pro‐fibrotic genes in liver tissue treated by dLSEC‐fpp was also downregulated compared with that treated by nLSEC‐fpp and vehicle controls, including *Acta2*, *Col1a1*, and *Col3a1* (Figure S29A–C, Supporting Information). Activated HSCs stained by *α*SMA also showed decreased expression in dLSEC‐fpp treated livers (Figure S29D, Supporting Information). In addition, no structural damage of other major organs besides livers could be found after the treatment (Figure S30, Supporting Information). Taken together, these results show that dLSEC‐fpp can effectively localize to fibrotic liver tissue in a long‐term treatment plan and alleviate ongoing advanced liver fibrosis.

### 2.7 dHUVECs Alleviate Advanced Liver Fibrosis in Mice and also Degrade Human Cirrhotic Scar Tissue

**Figure 7:**
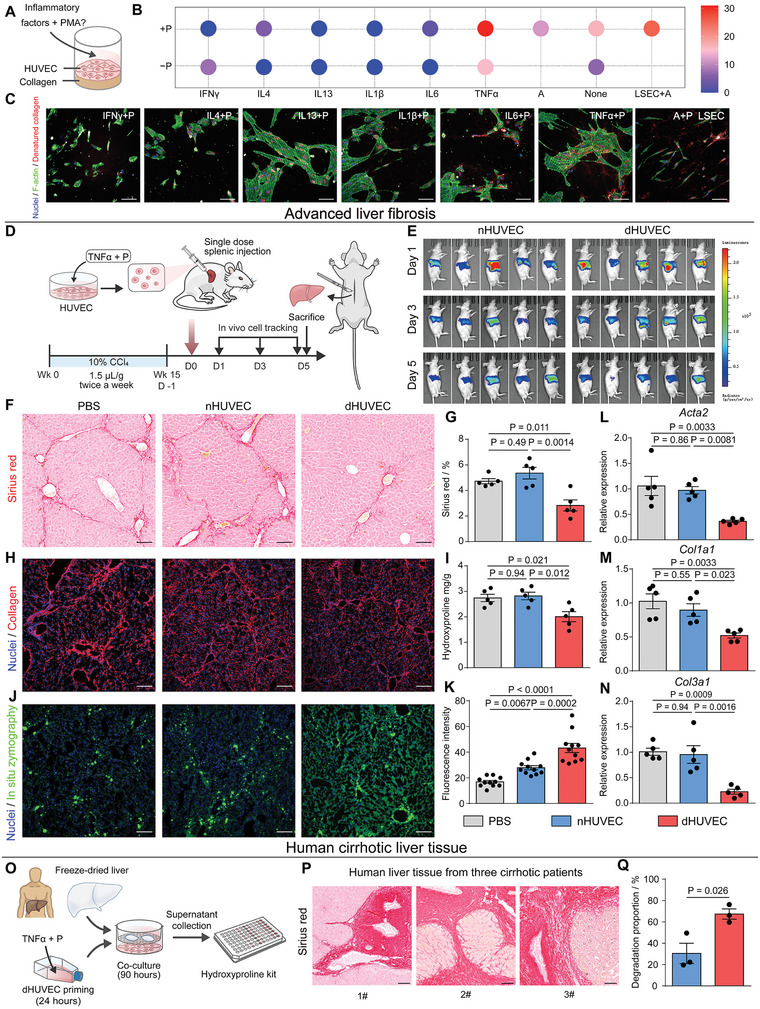
dHUVECs could alleviate the progression of advanced mouse liver fibrosis and degrade scar‐rich clinical samples from liver cirrhotic patients ex vivo. A) Schematic of constructing and characterizing ECM‐degrading HUVECs (i.e., dHUVECs) B) Collagen degradation ability of HUVECs with different stimulation strategies characterized by CEDSS. C) Representative high‐content fluorescent images showing the HUVEC‐mediated collagen degradation stained by CHP assay. Nuclei (blue), F‐actin (green), denatured collagen (red). Scale bars, 100 µm. D) Schematic of one‐dose intrasplenic administration of dHUVECs to treat the mice with advanced liver fibrosis induced by CCl_4_. E) Bioluminescent imaging of nHUVECs and dHUVECs resided in mice at day 1, 3, and 5 post injection F and G) Representative Sirius Red staining images and statistical analysis of collagen deposition in liver tissues treated by PBS, nHUVECs, and dHUVECs (*n* = 5, biological independent mice per group). Scale bars, 100 µm. H) Representative immunofluorescent images of collagen staining in liver tissues treated by PBS, nHUVECs, and dHUVECs. Scale bars, 100 µm. I) Quantification of hepatic hydroxyproline content. The data are expressed as hydroxyproline (mg) per liver dry weight (g) (*n* = 5, biologically independent mice per group). J and K) Representative images and statistical analysis of MMPs stained by in situ zymography in liver tissues treated by PBS, nHUVECs and dHUVECs (*n* = 11, points of detection derived from at least 3 biologically independent mice). Scale bars, 100 µm. L to N) Relative mRNA expression of *Acta2, Col1a1*, *and Col3a1* in liver tissues treated by PBS, nHUVECs and dHUVECs (*n* = 5, biologically independent mice per group). O) Schematic of characterizing the dHUVECs‐mediated degradation of scar‐rich clinical samples from liver cirrhotic patients ex vivo. P) Representative Sirius Red staining images of clinical liver samples from 3 patients with liver cirrhosis. Scale bars, 100 µm. Q) Statistical analysis of the dHUVECs‐mediated degradation of scar‐rich samples (*n* = 3, biological independent samples derived from 3 patients with liver cirrhosis). The statistical analysis in (Q) was performed using two‐tailed unpaired t‐test. The statistical analysis in (G, I, K–N) was performed using a one‐way ANOVA with Turkey test. Results are presented as means ± SEM.

In order to meet the requirement of clinical translation, we further examined the possibility of using an alternative cell type with comparable ECM degradation ability to dLSECs. By analyzing RNA‐seq data, we found that the differentially expressed genes in dLSECs were enriched in TNF signaling pathway (Figure 4C). Therefore, we hypothesized that inflammatory factors could potentially serve as alternatives to accutase, allowing us to construct cells with notable ECM degradation ability. We used CEDSS to characterize the ECM degradation ability of HUVECs, a type of cell widely used in clinical study,^[^ ^24^ ^]^ with stimulation by a series of inflammatory factors (**Figure** **7**A). Results showed that a stimulation strategy using a combination of tumor necrosis factor‐*α* (TNF*α*) and PMA could induce HUVECs with high ECM degradation ability (dHUVECs) which even exceeded that of dLSECs (Figure 7B). The high ECM degradation ability of dHUVECs was also confirmed by CHP staining through high‐content imaging (Figure 7C; Figure S31, Supporting Information). The ECM degradation ability of dHUVECs was maintained even after removal of TNF*α* and PMA factors after 24 h priming (Figure S32, Supporting Information). By comparing the gene expression of dHUVECs and normal HUVECs without stimulation (nHVUECs), we found an upregulation in *MMP1*, *9*, *10*, *14*, and *TIMP1*, and a downregulation in *MMP2*, *13*, *TIMP2*, and *TIMP4* in dHUVECs (Figure S33, Supporting Information). To validate the anti‐fibrotic effect of dHUVEC treatment in vivo, we established an advanced liver fibrosis murine model by CCl_4_ induction twice a week for 15 weeks, and then treated the fibrotic liver by using a one‐dose, intrasplenic administration of dHUVECs (Figure 7D). A number of dHUVECs could be detected in livers on the 5th day post injection (Figure 7E; Figure S34, Supporting Information). A decreased level of collagen deposition could be observed by Sirius Red staining and immunofluorescent staining in the liver tissue treated by dHUVECs compared with livers treated by nHUVECs and vehicle controls (Figure 7F–H). Consistently, the expression of hepatic hydroxyproline was the lowest in the dHUVEC‐treated group (Figure 7I). Results of in situ zymography assay also indicated an elevated expression of ECM‐degrading proteases in liver tissue treated by dHUVECs (Figure 7J,K). The expression of pro‐fibrotic genes in liver tissue treated by dHUVECs was also downregulated compared with livers treated by nHUVECs and vehicle controls, including *Acta2*, *Col1a1*, and *Col3a1* (Figure 7L–N). Activated HSCs stained by *α*SMA also showed decreased expression in dHUVEC treated livers (Figure S35, Supporting Information). Moreover, no structural damage of other major organs besides livers could be found after the treatment (Figure S36, Supporting Information). To further validate the potential of dHUVEC treatment to degrade the collagen‐rich scar tissue in clinical samples of cirrhotic liver tissue, we incubated the liver tissue from cirrhotic patients with dHUVECs ex vivo (Figure 7O,P). Results showed that a high proportion of cirrhotic liver tissue could be degraded by dHUVECs, which was ≈2.2 times higher than that degraded by nHUVECs (Figure 7Q). Interestingly, human decellularized cirrhotic liver tissue samples could also be effectively degraded by dHUVECs (Figure S37, Supporting Information). These results demonstrate the potential of dHUVECs for clinical treatment of liver cirrhosis.

## 3 Discussion

In some previous studies, fluorescence‐based assays have been used to visualize the cell‐mediated degradation of ECM, such as using fluorescence‐labeled gelatin‐coating substrate and CHP, a synthetic collagen‐like peptide that binds to deformed collagen peptide chains when the intact triple‐helix collagen structure is broken by the degradation effect.^[^ ^25^, ^26^ ^]^ However, precise quantification of ECM degradation ability of cells was impracticable to be assessed using these image‐based assays. Moreover, gelatin and synthesized peptide used in these assays could not recapitulate the properties of natural ECM in vivo. The CEDSS we constructed could precisely quantify the ECM degradation ability of different cells based on the characteristics of the fluorescent probe‐conjugated collagen matrix, which could be used to detect the degradation activity of various type of MMPs. Furthermore, other ECM components, in addition to type I collagen, could also be engineered in this system to better recapitulate the pathological ECM in cirrhotic liver tissue, such as elastin, fibronectin, and hyaluromic acid. In particular, elastin has been shown to dramatically increase at the late stage of liver fibrosis and is considered to be a critical pathological marker for liver cirrhosis.^[^ ^27^ ^]^ Therefore, constructing ECM to recapitulate the components of ECM in cirrhotic liver would be more helpful to screen out the candidates for liver cirrhosis treatment. It is also important to note that ECM in the cirrhotic liver is highly crosslinked, which results in increased resistance to protease‐mediated degradation. Some crosslinking characteristic of cirrhotic ECM, such as lysyl oxidase and transglutaminase‐mediated ECM crosslinking, should also be considered to further optimize our in vitro screening system.^[^ ^28^, ^29^ ^]^

Our results revealed a critical mechanism by which dLSECs mediated the degradation of ECM in vitro and in vivo through their secretion of MMPs (Figure 4 and Figure 6). In addition, the secretome of dLSECs also had the ability to deactivate HSCs in vitro (Figure S17, Supporting Information). Consistently, a lower proportion of activated HSCs was observed in liver tissues treated by dLSECs in liver fibrosis models in vivo (Figure S18, Supporting Information), which indicated that dLSEC treatment could alleviate advanced liver fibrosis through targeting HSCs along with MMP secretion. Taken together, our results indicated that dLSECs exerted a dual role on alleviating liver fibrosis, that is, degrading ECM through MMP secretion as well as inhibiting the pro‐fibrotic profile of HSCs. Furthermore, dLSECs could potentially inhibit HSCs activation in two different ways. First, dLSECs showed decreased level of pro‐fibrotic genes (i.e., TGFB2 and TGFB3) compared with nLSECs according to our RNAseq results, indicating their inhibitory effect on HSCs activation (Figure S38, Supporting Information). Secondly, collagen fiber‐mediated paratensile signaling has been reported to act as an essential mechanobiological way for HSCs activation during fibrotic liver diseases.^[^ ^30^ ^]^ Therefore, the degradation of collagen matrix by dLSECs could potentially block the paratensile‐induced HSCs activation, which benefit the alleviation of liver fibrosis.^[^ ^31^, ^32^ ^]^

In addition, our results showed that the ECM degradation ability of endothelial cells was regulated by c‐Fos‐mediated MMP expression (Figure 5F,J; Figure S13, Supporting Information). It was promising to manipulate the c‐Fos expression of endothelial cells near the fibrotic tissue by genetic approaches, such as mRNA delivery, which led to local release of MMPs to degrade the surrounding fibrotic tissue. This potential strategy could avoid the side effects associated with non‐specific ECM degradation effects caused by MMPs and generally improve clinical safety. Immune rejection is a critical issue that hinders the clinical application of cell therapy.^[^ ^33^ ^]^ In this study, we showed that dHUVEC treatment exhibited satisfactory therapeutic effects for advanced liver fibrosis in a SCID mouse model (Figure 7F–K). However, the feasibility of this approach for clinical translation needs to be further validated using animal models with a robust immune system.

Stem cell‐based therapies [e.g., mesenchymal stem/stromal cell (MSC)‐based therapy] have shown great potential in regenerative medicine to treat refractory diseases including liver fibrosis/cirrhosis.^[^ ^34^ ^]^ However, the huge gap between clinical and animal‐based pre‐clinical research in liver cirrhosis lies in the fact that the ECM scars in cirrhotic patients’ liver have been gradually formed in the period up to 30‐years, which are highly crosslinked and difficult to recover. Therefore, degrading the highly deposited scar, but not just inhibiting scar formation, could benefit cirrhotic patients. To best of our knowledge, few studies have reported the ECM‐degrading ability of MSCs, which is also validated by our CEDSS results, indicating the unsatisfactory ECM‐degrading ability MSCs (Figure 2C,D). In contrast, dLSECs showed great potential in degrading ECM in vitro, as well as alleviating late‐stage liver fibrosis (i.e., 15w CCl_4_‐induction) in vivo (Figures 3 and 6). In our ongoing work, we have found that embryonic stem cell‐derived dLSECs also possess the ability to degrade collagen matrix and could be further advanced for clinical applications (Figure S39, Supporting Information). Our results showed that the number of dLSECs was already relatively low 5 days after intra‐splenic injection (Figure S15, Supporting Information). In order to improve the effectiveness of cell therapy, further efforts are required to prolong the survival time of cells and promote cell growth and proliferation after administration. For example, it is possible to improve the survival of dLSECs by regulating the apoptosis suppressor gene *BCL‐2*,^[^ ^35^ ^]^ and thus improve the efficacy of treatment for chronic diseases such as liver cirrhosis. Since dLSECs could not be efficiently delivered to livers through intravenous injection (Figure S40, Supporting Information), intraperitoneal administration of dLSECs was applied in this study. Further research could focus on improving the targeting efficiency of intravenously injected dLSECs.

It is expected that ECM‐degradation therapy could be promising in clinical study. In addition, combinatory strategy with other cell therapy approach (e.g., MSC therapy) is expected to further strengthen the therapeutic efficacy of the dLSECs‐based therapy on liver cirrhosis, which might tune the immune microenvironment and promote liver regeneration.^[^ ^36^ ^]^ Considering the worldwide suffering from fibrotic diseases, which accounts for 45% of mortality in industrialized countries,^[^ ^30^ ^]^ our therapeutic strategy targeting deposited ECM in fibrotic tissues could also benefit a wide range of patients with fibrotic diseases, such as lung fibrosis, scleroderma, and kidney fibrosis.

## 4 Conclusion

Clinically, liver transplantation is still the only reliable treatment to prevent the deathly hurt caused by liver cirrhosis despite tremendous efforts and expenditure in therapeutics research and development.^[^ ^37^ ^]^ In this work, we constructed a CEDSS platform for high‐throughput detection of cellular degradation ability in vitro and found that combinatory priming with accutase and PMA could effectively endow LSEC with tremendous ECM degradation ability, which was regulated by MMP expression, downstream of transcription factor c‐Fos. In a CCl_4_‐induced advanced liver fibrosis mouse model, one dose intrasplenic administration of dLSECs promoted fibrosis recovery and systemic administration of liver‐targeted dLSECs showed therapeutic effects for alleviating progressive advanced fibrosis. A similar approach was used to construct dHUVECs with high ECM degradation ability, which promoted the recovery from advanced liver fibrosis in a mouse model and degraded ex vivo collagen‐rich liver tissue from liver cirrhosis patients. Our results provide a promising anti‐cirrhosis strategy by promoting degradation of scar tissue using ECM degradation‐based cell therapy.

## 5 Experimental Section

### Cell Culture

Human liver sinusoidal endothelial cells (LSECs) were a kind gift from L. Zhang's lab (Tsinghua University, Beijing, China), which were bought from ScienCell. Human hepatic stellate cell line (LX‐2) was bought from Xiangya Hospital of Centre‐South University. The acquirement and characterization of LSECs and LX‐2 cells were performed in the previous research.^[^ ^30^ ^]^ Mouse primary macrophages were isolated according to a published protocol.^[^ ^38^ ^]^ C166 cell line was purchased from American Tissue Culture Collection (ATCC) (CRL‐2581). Mouse macrophage cell line (Raw264.7) was purchased from ATCC (TIB‐71). These cells were cultured in Dulbecco's Modified Eagle Medium (DMEM) (Wisent, 319‐051‐CL) with 10% Fetal Bovine Serum (FBS) (Wisent, 086–150) and 1% penicillin–streptomycin (PS) (Wisent, 450‐201).

hESC‐derived MSCs and hESC‐derived LSECs were obtained and cultured according to published protocols.^[^ ^39^, ^40^ ^]^ For hESC‐derived LSECs, in brief, using self‐made AATS medium as basic medium, the embryonic stem cells were differentiated into mesodermal cells at 10% confluence. On the first day, 5 ng mL^−1^ BMP4 (Peprotech, 120‐05) and 10 µm CHIR (Peprotech, 100–18B‐250) were added, and 5 ng mL^−1^ BMP4 was added again on the second and third days. After treatment with 50 ng mL^−1^ VEGF (Peprotech, 100‐20‐250) and 10 ng mL^−1^ bFGF (Peprotech, 100–18B‐250) for three days, mesodermal cells differentiated to endothelial progenitor cells. Then FLK1^+^CD34^+^CD31^+^ liver sinusoid endothelial progenitor cells were sorted from endothelial progenitor cells by flow cytometry. On the basis of the medium used for the differentiation of endothelial progenitor cells, 5 µm TGF*β* inhibitor, SB431542 (selleck, S1067) was added for 5–7 days to promote the maturation of liver sinusoid endothelial cells. The characterization of hESC‐derived LSECs was performed by the positive staining of CD31, CD32, FVIII, LYVE1, and STAB2 markers (Figure S41, Supporting Information). For hESC‐derived MSCs, the embryonic stem cells were cultured in DMEM/F12 (Wisent, 319‐085‐CL) with 20% Knock‐Out Serum (Gibco, 10 828 010), *β*‐Mercaptoethanol (1000×, Gibco, 21 985 023), 10 ng mL^−1^ FGF2 (Novoprotein, C046), 1% GlutaMax (Thermo Fisher, 35 050 061), 1% non‐essential amino acids (NEAA, Gibco, 11 140 050), and 1% P/S (Gibco, 15 070 063) for three days. The medium was changed to 90% *α*‐MEM+GlutaMAX (Gibco, 41 090 036) with 10% Fetal Bovine Serum (Gibco, 10 091 148), 1% non‐essential amino acids (NEAA, Gibco, 11 140 050), 1% P/S (Gibco, 15 070 063), 10 ng mL^−1^ FGF2 (Novoprotein, C046), and 5 ng mL^−1^ TGF*β* (HumanZyme, HZ‐1011). After 10–15 days, cells were cultured in Human Mesenchymal Stem Cell Medium with 5% FBS, 1% mesenchymal stem cell growth supplements (ECGS) and 1% PS in plates coated with Matrigel (Corning, 354 277). After 4–5 days, cells were passed into plates coated with 0.1% Gelatin. Finally, CD73^+^CD90^+^CD105^+^ hESC‐derived MSCs were sorted by flow cytometry (Figure S42, Supporting Information).

Human primary umbilical vein endothelial cells (HUVECs) were purchased from China Center for Type Culture Collection (CCTCC). HUVECs were cultured in endothelial cell medium containing 5% FBS, 1% PS, and 1% endothelial cell growth supplements (ECGS) (ScienCell, SC‐1001‐prf).

Human monocytic cells (THP‐1) were cultured in RPMI 1640 Medium (Wisent, 350‐000‐CL) with 10% FBS and 1% PS.

Human adipose‐derived mesenchymal stem cells (ADMSCs) were obtained from Beijing CytoNiche Biotech. ADMSCs were cultured in Human Mesenchymal Stem Cell Medium with 5% FBS, 1% mesenchymal stem cell growth supplements (ECGS) and 1% PS.

Human promyelocytic cells (HL60) were purchased from National Institutes for Food and Drug Control (3111C0002000000098). HL60 were cultured in Iscove's Modification of DMEM (IMDM) (Wisent, 319‐112‐CL) with 20% Fetal Bovine Serum (FBS) and 1% penicillin–streptomycin (PS) (Wisent).

All the cells were cultured at 37 °C in a humidified atmosphere of 5% CO_2_ in air.

### Priming Cells

nLSECs were constructed by treating LSECs with DMEM medium containing 1% PS, 1× GlutaMax (Thermo Fisher, 35 050 061), and 0.5 mg mL^−1^ albumin (oryzogen, HYC002M01) for 24 h.

dLSECs were constructed by treating LSECs with DMEM medium containing 1% PS, 1× GlutaMax, 0.5 mg mL^−1^ albumin, 200× accutase (Thermo, A1110501), and 50 ng mL^−1^ PMA (Promega, V1171) for 24 h.

nHUVECs were constructed by treating HUVECs with ECM medium containing 1% PS, 1× GlutaMax and 0.5 mg mL^−1^ albumin for 24 h.

dHUVECs were constructed by treating HUVECs with ECM medium containing 1% PS, 1× GlutaMax, 0.5 mg mL^−1^ albumin, 40 ng mL^−1^ TNF*α* (Novoprotein, C008), and 50 ng mL^−1^ PMA for 24 h.

Cells screened by CEDSS were treated with corresponding basal medium as described above (DMEM for LSEC, LX2, C166, and Raw264.7, self‐made AATS medium for ESC‐LSEC, endothelial cell medium for HUVEC, RPMI 1640 Medium for THP1, Human Mesenchymal Stem Cell Medium for ADMSC and ESC‐MSC, IMDM for HL60) containing factors for 24 h. For groups without FBS, 1× GlutaMax and 0.5 mg mL^−1^ albumin were added in these groups. 200× Accutase, 50 ng mL^−1^ PMA, 2 µm fMLP (Sigma, F9758), 20 ng mL^−1^ LPS (Sigma, L3129), 20 ng mL^−1^ IL4 (Novoprotein, C050), 20 ng mL^−1^ IL13 (PeproTech, 200‐13‐10), 20 ng mL^−1^ TGF*β* (BioLegend, 781 802), 1.3% DMSO (Solarbio, D8371) or 40% FBS was added into medium for each group.

### Establishment of Cell‐Mediated ECM‐Degradation Screening System (CEDSS)

CEDSS comprises four major steps: 1) fluorescent collagen preparation; 2) cell seeding and priming; 3) supernatant collection; 4) characterization of degradation ability.

In the first step, 1 mL collagen solution was transferred into a 10 MWCO cut off dialysis bag. Dialysis was performed in pre‐cooled labeling buffer (0.25 m NaHCO_3_, 0.4 m NaCl, pH = 9.5) at 4 °C for 12 h. Then 1 mg NHS‐Rhodamine (Thermo, 46 406) was dissolved in 100 µL dimethylsulfoxide (DMSO) at room temperature and then diluted with 900 µL labeling buffer (filtered through a 0.22 µm membrane). The diluted NHS‐Rhodamine solution was added to the dialyzed collagen solution and the pre‐mixed solution was incubated at 4 °C in dark for 12 h with rotation. Then the mixture was dialyzed by pre‐cooled labeling buffer at 4 °C in dark for 12 h. The labeling buffer was replaced by pre‐cooled 0.2% acetic acid solution (pH = 4) every 12 h with continuous dialysis at 4 °C in dark. The Rhodamine‐modified collagen was then collected from the dialysis bag and stored at 4 °C in dark for the following study. To prepare Rhodamine‐modified collagen matrix, 19 µL DMEM basic medium was mixed with 0.6969 µL 1 m NaOH solution in ice bath followed by mixing with 27.55 µL unmodified collagen solution and 2.75 µL Rhodamine‐modified collagen matrix. The pre‐mixed solution was then transferred into a well in 96‐well plate and incubated at 37 °C for 100 min.

In the second step, 2 × 10^4^ cells were seeded into the prepared fluorescent collagen matrix with medium containing stimulating factors. In the third step, after 24 h or 48 h, the supernatant in the well was transferred to a light‐shielding 96‐well plate for the subsequent detection. In the last step, the fluorescent intensity of the collected supernatant, defined as F1, was detected at 575 nm emission and 546 nm excitation by a SpectraMax M5 multi‐well spectrophotometer (Molecular Devices, USA). The background intensity, defined as F2, was determined by the fluorescent intensity of culture medium. The intensity of complete‐degraded collagen matrix, defined as F3, was determined by absolute degradation of the collagen matrix by MMP1 (Gibco, 17 100 017), followed by detecting the fluorescent intensity of supernatant. The proportion of collagen degradation was determined according to Equation (1)

(1) $$\mathrm{collagen}\;\mathrm{degradation}=\frac{F1-F2}{F3-F2}*100\%$$

### High‐Content Imaging

Cells grown on collagen matrix were fixed by 4% paraformaldehyde (PFA) for 4 h and then washed by phosphate‐buffered saline (PBS) every 30 min for four times. CHP (Advanced biomatrix, 5276) staining was performed according to the manufacturer's instructions. Nuclei were stained by Hoechst 33 342 (Beyotime, 1:1000) for 30 min and Filamentous actin (F‐actin) was labelled by Acti‐stain^TM^ 488 phalloidin or Acti‐stain^TM^ 555 phalloidin (Cytoskeleton, 1:400) for 30 min. Then the samples were washed by PBS every 30 min for four times. High‐content imaging was performed by Operetta (Perkin Elmer, USA). The samples were also imaged by Leica SP8 confocal microscope to characterize the interactions between cells and collagen fibrils.

### Immunofluorescence Staining

Cells or cells growing on collagen were fixed by 4% PFA and then washed three times with PBS. The samples were permeabilized and blocked by PBS containing 0.3% Triton X‐100 (Sigma, T8787) and 3% bovine serum albumin (BSA) (Amresco) for 1 h. The following primary antibodies were used: anti‐c‐Fos (Abcam, ab190289), anti‐MMP9 (Thermo, MA515886), anti‐Paxillin (Abcam, ab32115), anti‐collagen IV (Abcam, ab6586), anti‐CD31 (Abcam, ab9498), anti‐CD32 (Abcam, ab131051), anti‐LYVE1 (Abcam, ab14917), anti‐FVIII (Abcam, ab275376), and anti‐STAB2 (Abcam, ab121893). Cells were washed three times with PBS. The fluorescent secondary antibody was incubated for 1 h. Cells were washed three times with PBS.

### Ponceau S Staining of Collagen

Collagen was fixed by 2.5% glutaraldehyde for 30 min. The glutaraldehyde fixative was carefully removed and stained with 0.1% Ponceau S red (Beyotime, P0022) for 30 min. Samples were washed three times with deionized water to remove floating colors.

### Scanning Electron Microscopy

The samples were fixed by PFA for 2 h and then washed by deionized water for 4 h. Dehydration was carried out in a gradient of 50%, 70%, 80%, 90%, and 100% ethanol. Samples were dried using CO_2_‐critical‐points‐drying methods by Leica EM CPD300 and then coated with gold for 90 s. Images were taken by a FEI Quanta 200 scanning electron microscope.

### Tube Formation Assay

Matrigel(60 µL) was added to a 35‐mm‐diameter confocal dish (In Vitro Scientific, D35‐10‐1‐N). 2 × 10^4^ nLSECs or dLSECs were seeded on the Matrigel with 100 µL DMEM medium containing 1% PS, 1× GlutaMax (Thermo Fisher, 35 050 061), and 0.5 mg mL^−1^ albumin (oryzogen, HYC002M01). After 8 h, the medium was discarded and 100 µL Calcein AM (2000×, diluted with PBS) (KGAF001, KeyGen BioTECH) was added to the dish. After incubation at 37 °C for 15 min, the dye was discarded and cells were washed twice with PBS. The images were taken by Olympus FV3000 confocal laser scanning microscope and Image J with the Angiogenesis Analyzer plugin was used for the quantification of tube networks.

### Secretome Analysis

nLSECs and dLSECs were incubated in RPMI 1640 basic medium for 24 h. Then the medium was collected and centrifuged at 1500 rpm for 5 min. The supernatant was retained and filtered with a 0.45‐µm filter. Pre‐cooled acetone was added to the supernatant at a ratio of 5:1 at −20 °C (acetone to supernatant, % v/v). After standing at −20 °C for 4 h, the solution was centrifuged at 7500 rpm for 20 min. The supernatant was discarded and the pellet was dried naturally. The pellet was dissolved with protein lysis buffer (PBS containing 8 M urea). The concentration of protein was determined using the BCA Protein Assay Kit (Beyotime, P0010S). Proteins were first reduced with dithiothreitol (Sigma‐Aldrich, D9760) and alkylated with iodoacetamide (Sigma‐Aldrich, I6125), then digested into peptides by sequencing‐grade trypsin (Promega, V5111). After desalting with Sep‐Pak C18 (Waters) and labelling with tandem mass tag reagent (Thermo Fisher Scientific), the final peptides were divided into 12 fractions by the first‐dimension reversed‐phase liquid chromatography.

Online‐coupled nano–high‐performance liquid chromatography system (Thermo Fisher Scientific) and Orbitrap Fusion Lumos mass spectrometer (Thermo Fisher Scientific) were used for liquid chromatography‐tandem mass spectrometry (LC‐MS/MS). With mobile phase A (0.1% formic acid in water) and mobile phase B (0.1% formic acid in acetonitrile), the polypeptide samples were gradient‐eluted in elution buffer at a flow rate of 0.25 µL min^−1^ for 2 h. The data‐dependent acquisition mode of the Xcalibur software (version 3.0) was fixed, and then a full‐scan mass spectrum was acquired. Data‐dependent MS2 scans were performed at higher‐energy collisional‐based fragmentation 20 times. Data from each LCMS/MS run were examined together with the UniProt database through Proteome Discoverer software (version 2.1, Thermo Fisher Scientific).

### Preparation of Mouse Liver Decellularized Matrix

Mice were anesthetized with 2.5% avertin. The abdomen of mice was opened and the indwelling needle was inserted into their inferior vena cava. Deionized water (ddH_2_O) was perfused with a peristaltic pump until the liver swelled. The portal vein was cut and the inferior vena cava was ligated to secure the indwelling needle. A peristaltic pump was used to perfuse the liver with ddH_2_O for 30 min, 0.01% SDS solution for 2 h, 0.1% SDS solution for 2 days, and ddH_2_O for 12 h in order. The liver decellularized matrix was cut out and washed in ddH_2_O on a shaker overnight (During this period, ddH_2_O was replaced several times). Then, matrices were dehydrated with 50%, 70%, 80%, 90%, and 95% ethanol on a shaker for 10 min in order. and dehydrated three times with absolute ethanol for 10 min on a shaker. After CO_2_ critical point drying, matrices were stored under a low vacuum.

### Human Liver Cirrhotic Specimens

The fresh liver tissues from patients with established liver cirrhosis (patients 1#, 2#, 3#) were provided by the Department of Hepatology at Tsinghua Changgung Hospital and approved by the Institutional Review Board of Tsinghua (Project No: 20 210 164). Informed written consent was obtained from all participants. The extent of liver cirrhosis was verified by Sirius Red staining. The clinical liver samples were inactivated at 110 °C for 30 min and then cut into small pieces of appropriate size. The surface of samples was dried with filter paper. The samples were lyophilized after being frozen at −80 °C and sterilized with ethylene oxide.

### Hydroxyproline (HYP) Assay

3 × 10^5^ dLSECs or dHUVECs were cultured in the bottom well in 12 mm transwell plate (Corning, 3462) and dried mouse decellularized matrix or lyophilized human cirrhotic liver tissues were placed in the insert well with 3.0 µm pore polyethylene terephthalate (PET) membrane. The samples were incubated at 37 °C for 90 h. The HYP content of supernatant in insert well (H1), of supernatant in bottom well (H2), and of remaining samples in insert well (H3) were detected by Hydroxyproline (HYP) Content Assay Kit (Solarbio, BC0255) according to the manufacturer's instructions. The proportion of sample degradation was determined according to the Equation (2)

(2) $$\mathrm{Sample}\;\mathrm{degradation}=\frac{H1+H2}{H1+H2+H3}*100\%$$

The HYP content of liver tissue samples were also characterized Hydroxyproline (HYP) Content Assay Kit (Solarbio, BC0255) according to the manufacturer's instructions.

### RNA Isolation and Quantitative Real‐Time PCR (qPCR)

Livers were homogenized in Total RNA Extraction Reagent (Vazyme, R401‐01). For isolating RNA from cells, cells were treated by total RNA extraction reagent. 1/5 volume of chloroform was added into lysate and the mixture was centrifuged at 12 000 g for 15 min at 4 °C. The upper aqueous phase was collected and mixed with an equal volume of isopropanol. Then the mixture was cooled at 4 °C for 10 min and centrifuged at 12 000 g for 15 min at 4 °C. The precipitate was washed twice with 75% ethanol. The precipitate was resuspended in DNase/RNase‐Free Water (Solarbio, R1600) after drying in air. RNA concentration and purity (A_260/280_ and A_260/230_) were measured by NANODROP 2000 (Thermo). cDNA was synthesized from 1 µg RNA using a Hiscript II qRT SuperMix Kit (V) (Vazyme, R222‐01) in a total volume of 20 µL in T100^TM^ Thermal Cycler (Bio‐Rad). Reverse transcription conditions were as follows: 50 °C for 15 min followed by 85 °C for 5 s.

Expression of different genes were characterized using quantitative real‐time PCR in triplicate with in a CFX96 machine (Bio‐Rad). cDNA(0.4 µL), 0.5 µL of forward primer, 0.5 µL of reverse primer, 3.6 µL of DNase/RNase‐Free Water, and 5 µL of AceQ qPCR SYBR green master mix (Vazyme, Q121‐02) were added into the reaction. PCR conditions were as follows: 95 °C for 5 min followed by 40 cycles of 95 °C for 15 s and 60 °C for 30 s. Primers used were listed as in Table S2 (Supporting Information) with *GAPDH* as the reference genes. Values were normalized to *GAPDH*, and data are shown as fold differences of 1 from the reference sample set (2^−ΔΔCT^).

### Matrix Metalloproteinase (MMPs) Array

Supernatants of dLSECs and nLSECs culturing medium were collected after 2 days’ culture. The expression of MMP‐1, MMP‐2, MMP‐3, MMP‐8, MMP‐9, MMP‐10, MMP‐13, Tissue inhibitor matrix metalloproteinase ‐1 (TIMP‐1), TIMP‐2, and TIMP‐4 were detected by human MMPs array kit (Raybiotech, QAH‐MMP‐1) according to the manufacturer's instructions. Fluorescent signals were scanned by Agilent SureScan Dx Microarray Scanner and GenePix Pro 6.0 software (Axon Instruments, USA) was used to analyze the data.

### RNA Sequencing

RNA sequencing analysis was performed on LSECs and conducted by Biomarker Technology Corporation (Beijing, China). Specifically, total RNA of LSECs in vitro was extracted by using TRIZOL (Vazyme) reagent. A total amount of 1 µg RNA was used for library construction and sequenced by using VAHTS Universal V6 RNA‐seq Library Prep Kit for Illumina ® (NR604‐01/02). Around 40 million and 45 million reads were obtained for nLSECs sample and dLSECs sample, respectively. Clean reads were mapped to the human genome sequence (http://ftp.ncbi.nlm.nih.gov/genomes/refseq/vertebrate_mammalian/Homo_sapiens/latest_assembly_versions/) using STAR. Differentially expressed genes were quantified using DESeq. The genes with counts greater than 10, log_2_‐transformed fold change > 1, and *q* values <0.01 were considered to be differentially expressed. Data post‐processing and visualization were performed in R (Version 4.1.2). ClusterProfiler package (Version 4.4.4) was used to perform functional enrichment for Gene Ontology (GO) and Kyoto Encyclopedia of Genes and Genomes (KEGG). Heatmaps were plotted based on logarithmic transformation of FPKM (Fragments Per Kilobase per Million) which was log_10_ (FPKM+1) for genes under each experimental group using ggplot2 (Version 3.3.6) and pheatmap (Version 1.0.12). The raw sequencing data were available in SRA database under the accession number SRR21982014 and SRR21983413.

### KEGG Analysis

Differentially expressed genes in dLSEC group compared with nLSEC were filtered based on the FDR which is lower than 0.05. Gene symbol was further transformed to ENTREZID according to the org.Hs.eg.db (Homo sapiens). KEGG analysis was performed on genes with significant variations in expression through enrichKEGG (clusterProfiler (version 3.0.4)) to determine enriched high‐level biological process where pvaluecutoff, minGSSize and maxGSSize were respectively set to 0.05, 5, and 500. Results were visualized by dotplot where 10 pathways with the lowest *p* value were depicted and the size of the dot indicates the number of enriched differentially expressed genes while the color shows the level of adjusted *p* value.

### Confocal Imaging and Analysis

Confocal images were taken using Olympus FV3000 microscope. Images were captured using ×100 oil objective or × 40 air objective. Nuclear level of expression was defined as the sum intensity of c‐Fos in nuclear area, and nuclear / cytoplasm ratio was defined as the ratio of nuclear expression level to the cytoplasm expression level in a cell. MMP9 expression was defined as the sum intensity in a cell. Imaging conditions were kept the same in experiments for comparative analysis. All of the quantitative analyses were carried out using Imaris 9.6.0 with consistent setups.

### Inhibiting c‐Fos with T5224

To inhibit c‐Fos, LSECs were treated with DMEM medium containing 1% PS, 1× GlutaMax, 0.5 mg mL^−1^ albumin and 100 µm T5224 (Selleck, S8966) in nLSEC+T5224 group. Correspondingly, LSECs were treated with DMEM medium containing 1% PS, 1× GlutaMax, 0.5 mg mL^−1^ albumin, 200× accutase, 50 ng mL^−1^ PMA, and 100 µm T5224 in dLSEC+T5224 group. At particular time points after stimulating treatment (i.e., 3 h for c‐Fos and 24 h for MMPs), cells were subjected to Total RNA Extraction Reagent for quantitateve PCR analysis or fixed by 4% PFA for immunofluorescence staining. And cells were lysed with radio immunoprecipitation assay buffer for western blot or seeded oto the prepared fluorescent collagen matrix for characterization of degradation ability as described 24 h after treatment.

### Construction of LSEC‐Akaluc and HUVEC‐Akaluc

LSECs or HUVECs used for in vivo experiments were engineered to stably express mVenus and Akaluc by lentiviral mediated gene transfer (LSEC‐Akaluc and HUVEC‐Akaluc). Plasmids and recombinant cells were constructed according to the published protocol.^[^ ^41^ ^]^

In brief, for lentiviral production, the plasmid vectors (5 µg pVSV‐G, 10 µg pΔ8.9, 15 µg pLV‐CMV‐mVenus‐Akaluc) were diluted and mixed with 100 µL opti‐MEM, followed by the addition of 30 µL Neofect (Neofect biotech). The mixture was added to the cell medium and mixed gently. Supernatant was collected through centrifugation at 19 500 rpm for 2.5 h at 4 °C after 72 h transfection. The virus suspension was obtained by resuspending the precipitate with 200 µL opti‐MEM.

For viral infection, the suspension was added to the 6‐well plate seeded with cells (LSECs or HUVECs). Polyberene (8 µg mL^−1^) was added to facilitate the viral infection. Medium was changed after 24 h. When the cells were passaged to a certain number, the successfully infected cells were selected by flow sorting (BD Influx).

### Bioluminescence Monitoring of LSEC‐Akaluc and HUVEC‐Akaluc

For in vivo tracking of LSECs or HUVECs, cells were stably transfected to express Akaluc by lentiviral mediated gene transfer (LSEC‐Akaluc or HUVEC‐Akaluc). To track the distribution of LSEC‐Akaluc or HUVEC‐akaluc in vivo, mice were intraperitoneally injected with 40 µL of TokeOni (15 mg mL^−1^, 808 350, Sigma‐Aldrich) at 10 min before imaging using an IVIS Spectrum imaging system (PerkinElmer).

### Animal Models

Balb/c nu mice were purchased from the Laboratory Animal Resources Center at Tsinghua University, which was accredited by Association for Assessment and Accreditation of Laboratory Animal Care International. All animal protocols of this study were approved by Institutional Animal Care and Use Committee (IACUC) of Tsinghua University. For all in vivo experiments, mice were randomly and blindly divided into different groups. There were at least four biologically independent mice per group.

For carbon tetrachloride (CCl_4_)‐induced liver injury model, 6‐ to 8‐week‐old nude male mice were injected intraperitoneally with CCl_4_ (5 ml kg^−1^, 1:4 mixtures of CCl_4_ and olive oil) 24 h before dLSEC‐fpp injection. For CCl_4_‐induced advanced liver fibrosis model, 6‐ to 8‐week‐old nude male mice were intraperitoneally injected with CCL_4_ (1.5 ml kg^−1^, 1:9 mixtures of CCl_4_ and olive oil) twice a week for 15 weeks or 20 weeks.

### Targeting Efficiency of dLSEC‐fpp in a Model of Acute Liver Injury

1 × 10^6^ dLSECs or dLSEC‐fpp in 100 µL PBS were intraperitoneally injected into mice with acute liver injury. The organs of treated mice were harvested for anatomical imaging on day 3 after injection to observe the distribution of dLSECs or dLSEC‐fpp.

### Anti‐Fibrosis Treatments

For one‐dose dLSEC or dHUVEC therapy, 4 × 10^5^ LSECs (dLSECs or nLSECs) and HUVECs (dHUVECs or nHUVECs) in 100 µL PBS were intrasplenically injected into mice with established fibrosis using a syringe pump as described previously.^[^ ^23^ ^]^ Cell distribution in mice was imaged on day 1, 3, and 5 post‐injection. The mice were sacrificed and the livers were harvested on day 5 of post‐treatment.

For systemic administration, advanced liver fibrosis was established by 20‐week induction using CCl_4_. 1 × 10^6^ dLSEC‐fpp or nLSEC‐fpp in 100 µL PBS were intraperitoneally injected into mice with established advanced liver fibrosis 48 h after the CCl_4_ injection at week 20. Then the administration was repeated at day 9 and day 16 after the initial administration as illustrated in Figure 6I. CCl_4_ was injected routinely during this time to keep the progression of liver fibrosis. The mice were euthanized at day 21 and the livers were collected for subsequent assays.

### Histological Staining

Frozen sections(7 µm‐thick) were fixed with 4% PFA for 30 min, and then washed by PBS for three times. The samples were permeabilized and blocked by PBS containing 0.3% Triton X‐100 (Sigma) and 3% bovine serum albumin (BSA) (Amresco) for 1 h. The primary antibody was incubated overnight at 4 °C. The following primary antibodies were used: anti‐COL‐1 (Abcam, ab6308), anti‐*α*SMA (eBioscience, 14‐9760‐82), anti‐F4/80 (Abcam, ab6640), anti‐CD80 (Biolegend, 104 705), anti‐Arg1 (Cell Signaling Technology, 93 668), anti‐CD31 (Abcam, ab9498), anti‐Ki67 (eBioscience, 12‐5698‐82), and anti‐ALB (Abcam, ab207327). The samples were incubated with fluorescent secondary antibody for 1 h at room temperature. The samples were washed by PBS for three times. Collagen was stained by Sirius Red staining (Huayueyang Bio‐Technology, GH6044s) according to manufacturer's instructions. In situ zymography was performed by using in situ zymography kit (Genmed, GMS80095.1) according to manufacturer's instructions. Histological images were taken using a microscope (3DHISTECH Pannoramic SCAN) or a confocal laser scanning microscope (Olympus FV3000). Image analysis was carried out using Imaris 9.6.0 with a consistent setup.

### Cell Membrane Modified Functional Peptide (fpp)

Liver‐targeting peptides (fpps, GQLKHLEQQEG)^[^ ^23^ ^]^ were dissolved in DMEM medium containing 1% PS, 1× GlutaMax (Thermo Fisher, 35 050 061), and 0.5 mg mL^−1^ albumin (oryzogen, HYC002M01). To optimize the conditions of peptide modification, fpps with different concentrations (0.01 mm, 0.1 mm, 1 mm, 5 mm, and 10 mm) were incubated with cells for different modification time (5 min, 10 min, 20 min, 30 min, 40 min, 50 min, and 60 min). Fpp solution was sterilized with a 0.22‐µm membrane filter. 1 × 10^6^ cells were incubated with 100 µL peptide solution at 37 °C to allow the modification of fpps on cell membrane. Then, cells were washed with PBS and used for subsequent experiments. LSECs modified by FITC‐labeled fpps were imaged using a confocal laser scanning microscope (Olympus FV3000).

### Preparation of Liver Homogenates and Determinations of Inflammatory Cytokines

Liver tissue was washed with pre‐cooling PBS to remove blood on the surface. Liver tissue was dried with filter paper three times, weighed and transferred to 1.5 mL polypropylene tubes. Lysis buffer was prepared by adding protease inhibitors (100×) (Abcam, ab270055) to pre‐cooling PBS. Every 100 mg tissue was added to 500 µL of lysis buffer. Samples were homogenized with an electrical homogenizer (PRO Scientific, Bio‐Gen PRO200) until no macroscopic tissue was observed. The homogenates were centrifuged at 10000 ×g for 10 min at 4 °C. The supernatant was collected and the concentration of protein was determined using the BCA Protein Assay Kit (P0010S, Beyotime) according to the manufacturer's instructions. The concentration of inflammation‐involved factors such as interleukin‐2 (IL‐2), IL‐6, interferon‐*γ* (IFN*γ*), and tumor necrosis factor‐*α* (TNF*α*) concentrations were determined in liver homogenates using Luminex® xMAP® technology (eBioscience, EPXS050‐22199‐901) following manufacturer's instructions. Data were normalized to liver content and expressed as ng g^−1^ of protein.

### Western Blot

Cells were lysed with radio immunoprecipitation assay buffer (Leagene, PS0012). Then, the concentration of protein was determined using the BCA Protein Assay Kit (Beyotime, P0010S). Protein samples mixed with SDS‐PAGE Protein Loading Buffer (5 ×) (Beyotime, P0015L) were heated at 100 °C for 10 min. Protein samples (20 µg) were separated by 10% SDS‐polyacrylamide gel and transferred to a polyvinylidene fluoride membrane (Millipore, IPVH00010). Membranes were blocked with 5% nonfat dried milk (FUJIFILM, 190–12865) for 1 h and then incubated with primary antibody (anti‐*β*‐actin, 1:5000, Cell Signaling Technology, 4970S; anti‐MMP9, 1:900, Invitrogen, MA5‐15886) diluted with primary antibody dilution buffer (Solarbio, A1810) overnight at 4 °C. Membranes were washed with TBST buffer (Biosharp, BL315B) and incubated with secondary antibodies (Goat Anti‐Rabbit IgG, 1:4000, ABclonal, AS014; Goat Anti‐Mouse IgG, 1:4000, EASYBIO, BE0102) diluted with 5% nonfat dried milk for 1 h at room temperature. Images were acquired by Bio‐Rad ChemiDoc MP (Bio‐Rad).

### Transmission Electron Microscopy (TEM)

TEM samples were prepared as described previously.^[^ ^42^ ^]^ Briefly, cells growing on collagen samples were fixed in 2% PFA‐2.5% glutaraldehyde (Structure Probe, SPI‐CHEM, 02607‐BA) fixative solution (pH = 7.2) for 1 h. The samples were washed by PBS for four times. 1% OsO4 (Ted Pella, 18 451)‐1.5% Potassium hexacyanoferrate(II) trihydrate (Sigma‐Aldrich, P3289) fixative solution was used to fix the samples for 30 min. Then the samples were washed by PBS followed by being washed by deionised water for 3 times. 1% uranyl acetate (Structure Probe, SPI‐CHEM, 1 161 108) was used to stain the samples in dark at room temperature for 1 h. Then the samples were washed by deionized water for four times. Dehydration was carried out in a gradient of 50%, 70%, 80%, 90% ethanol (Sinopharm Chemical Reagent, 10 009 218) and 100% alcohol for three times with each dehydration lasted for 2 min. Samples were incubated with epon 812 (Structure Probe, SPI‐CHEM, 02659‐AB): ethanol at the volume proportion of 1:1 for 2 h, 2:1 for 4 h, and 3:1 overnight at room temperature. Then, samples were incubated with epon 812 at room temperature 2 times for 8 h each. Resin was aggregated at 60 °C for 24 h. The embedding block was cut into 80‐nm ultrathin sections on ultramicrotome (Leica Microsystems, model: EM UC6) with a diamond knife and collected by Formvar‐coated grids (Emcn, AZH75HH). Sections were stained by uranyl acetate for 30 min and lead citrate (Lead acetate: Structure Probe, SPI‐CHEM, 1 161 108, Sodium Citrate, 98%+: damas‐beta, 76198A) for 5 min. Images were taken by transmission electron microscope (Hitachi High‐Technologies, H‐7650).

### Statistics

Statistical analysis was performed by GraphPad Prism 6. A two‐tailed unpaired Mann Whitney test or unpaired *t*‐test was used when comparing two groups of data, and ANOVA following Tukey's multiple comparisons test was used when comparing three or more groups of data. All data are presented as mean values ± SEM as indicated in figure legends and *p*‐values are marked in the figures.

## Conflict of Interest

The authors declare no conflict of interest.

## Author Contributions

P.Z. and Y.D. conceived and designed the researches. P.Z. designed and established CEDSS system, performed mechanism study, and analyzed and interpreted data. P.Z., T.S., and C.C. conducted animal experiments. C.L. helped with the mechanism study. C.X. provided clinical samples and helped with the clinical consultation. K.L. prepared the schematics. Y.N. analyzed the data of RNA‐seq and MMPs array. Y.Z. provided the hESC derived LSECs. P.Z. and Y.D. wrote the manuscript. Y.D. is the principal investigator of the supporting grants.

## Supporting information

Supporting Information

Supplemental Movie 1

Supplemental Movie 2

## Data Availability

The data that support the findings of this study are available in the supplementary material of this article.
