# Supplementary material for: Scar‐Degrading Endothelial Cells as a Treatment for Advanced Liver Fibrosis
Source: Adv Sci (Weinh). 2022 Dec 9;10(4):2203315. doi: 10.1002/advs.202203315 (PMC9896053; doi:10.1002/advs.202203315)
Supplement: Supplementary file 1 — Supporting Information [file ADVS-10-2203315-s001.pdf]

ADVANCED SCIENCE
Open Access

Supporting Information

for *Adv. Sci.*, DOI 10.1002/advs.202203315

Scar-Degrading Endothelial Cells as a Treatment for Advanced Liver Fibrosis

*Peng Zhao, Tian Sun, Cheng Lyu, Kaini Liang, Yudi Niu, Yuying Zhang, Chenhui Cao, Canhong Xiang and Yanan Du**

Scar-Degrading Endothelial Cells as a Treatment for Advanced Liver Fibrosis

Authors: *Peng Zhao, Tian Sun, Cheng Lyu, Kaini Liang, Yudi Niu, Yuying Zhang, Chenhui Cao, Canhong Xiang, Yanan Du**

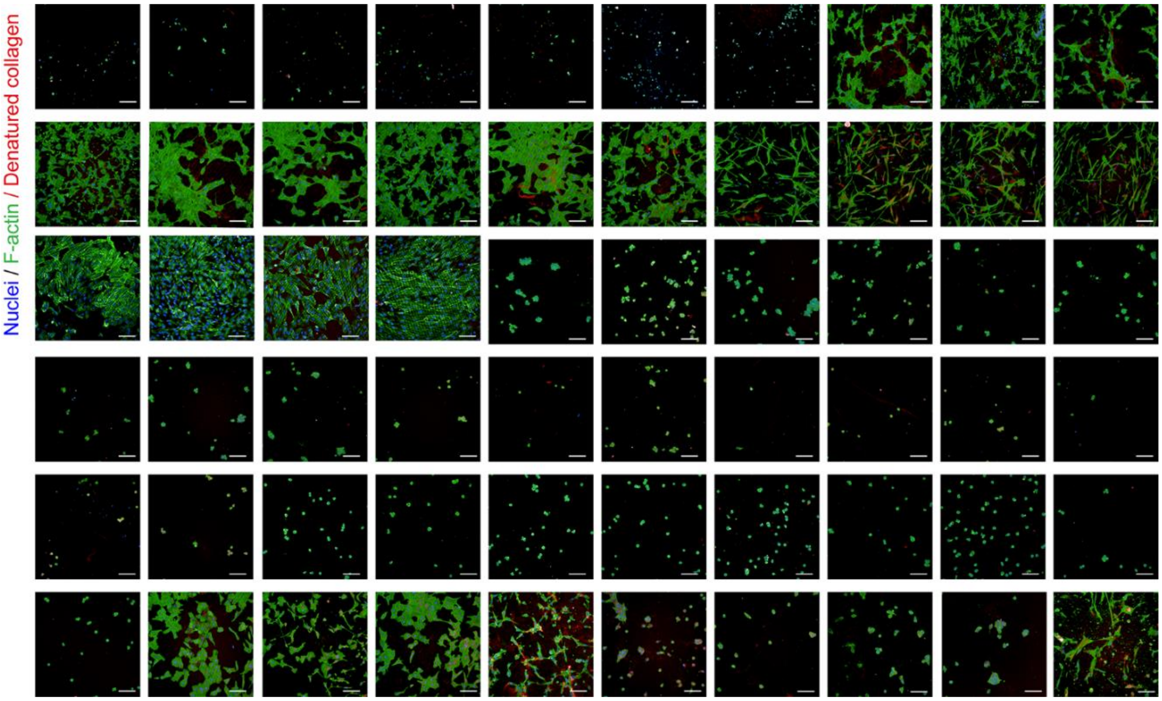

Figure S1. High-content fluorescent images showed the cell-mediated collagen degradation stained by CHP assay. Detailed settings of experiments could be referred to Supplementary table 1 and experimental section (priming cells). Representative high content images of collagen degradation stained by CHP assay in different cells stimulated by different combinations. Nuclei (blue), f-actin (green), denatured collagen (red). Scar bar, 100 μm.

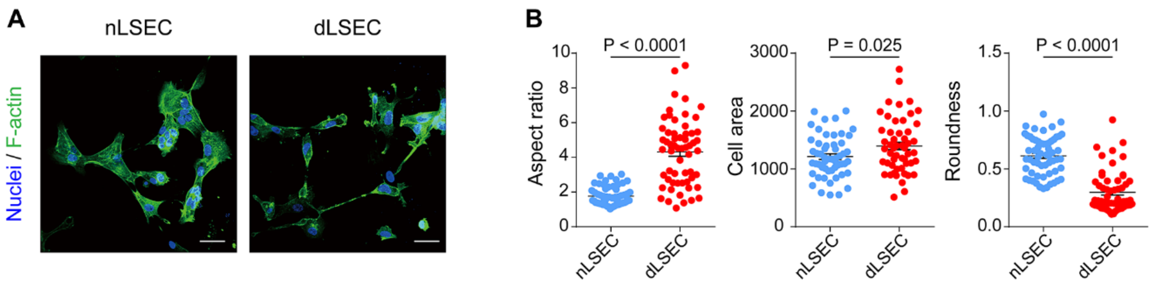

Figure S2. The morphologies of dLSECs were significantly different from nLSECs.
(**A**) Representative fluorescent images showing the F-actin staining of nLSECs and dLSECs. Scale bars, 40 μm. (**B**) Statistical analysis of aspect ratios, cell area, roundness of nLSECs and dLSECs (n > 50, number of cells analyzed per condition). Statistical analysis was performed using two-tailed unpaired t-test. Results are presented as means ± SEM.

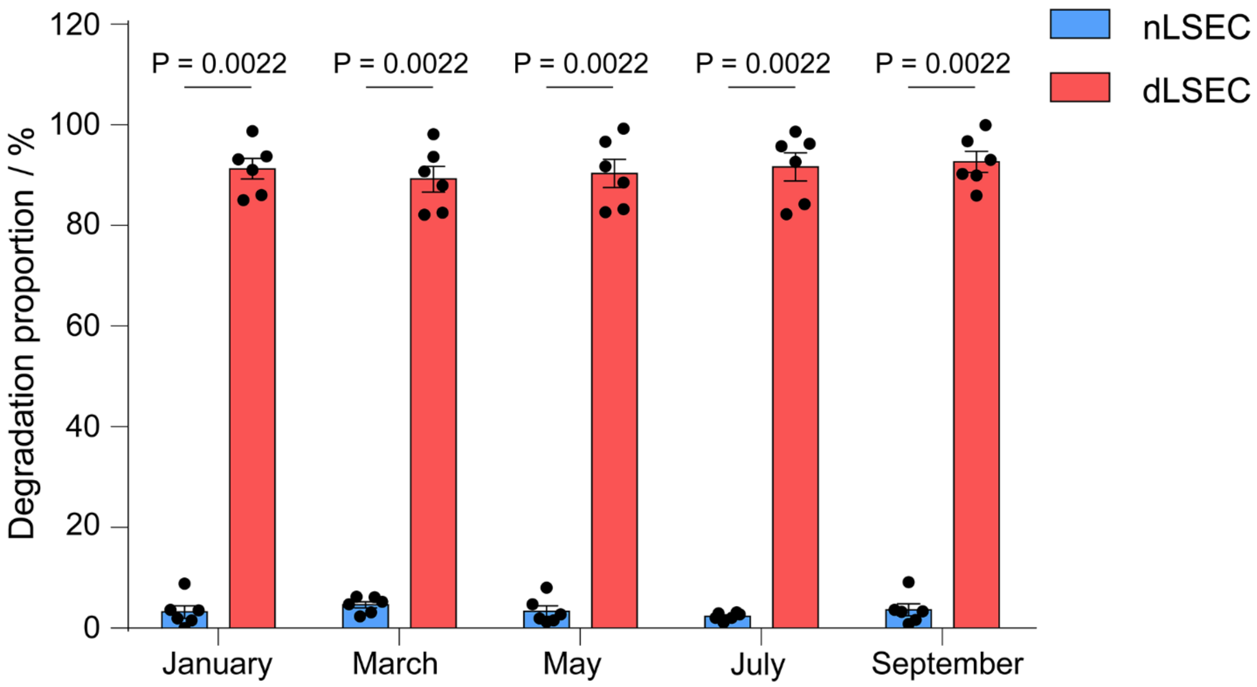

Figure S3. Repetitive validation of dLSECs-mediated ECM degradation by using CEDSS test. The experiments were performed by at least two independent researchers at different time points within a year. Statistical analysis was performed using two-tailed unpaired t-test. Results are presented as means ± SEM.

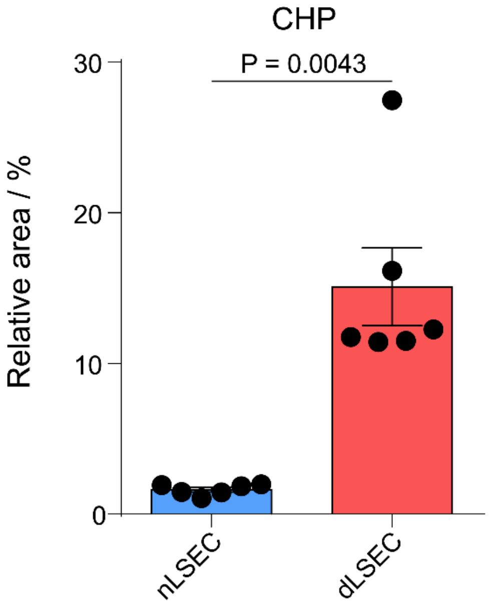

Figure S4. A strong CHP signal could be observed in the area surrounding the dLSECs compared with nLSECs. Statistical analysis of CHP staining of collagen matrix with degradation mediated by nLSECs and dLSECs. (n = 6, biological independent samples). Statistical analysis was performed using two-tailed unpaired t-test. Results are presented as means ± SEM.

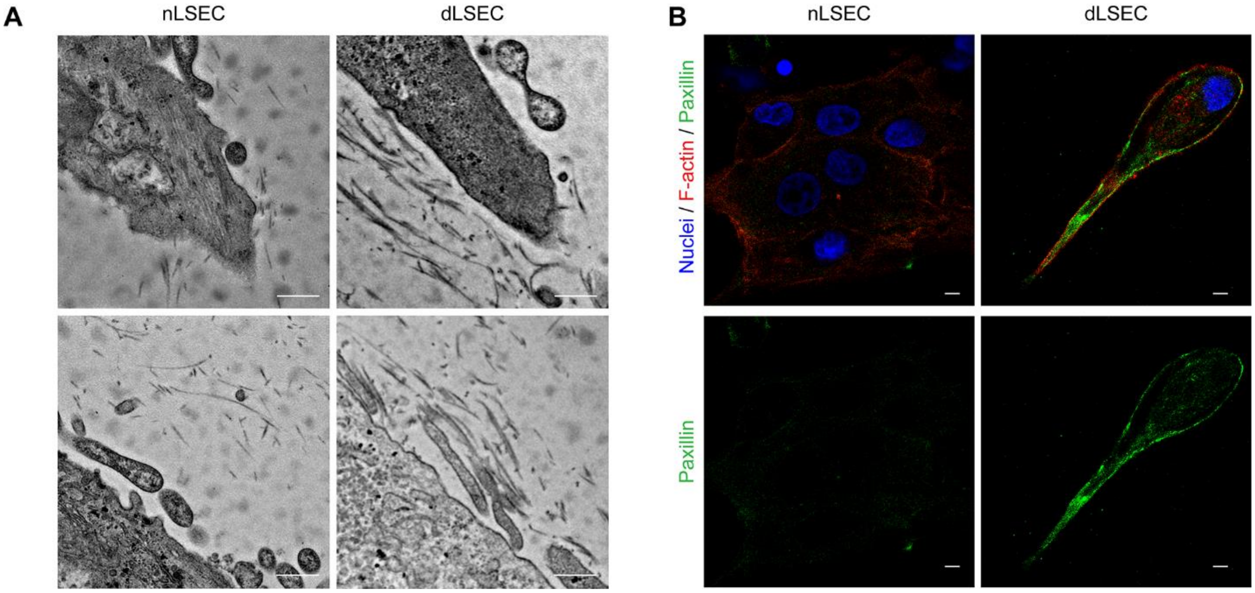

Figure S5. dLSECs grown on collagen matrix showed increased cell-matrix interactions compared with nLSECs. (**A**) Representative TEM images of nLSECs and dLSECs grown on collagen matrix. Scale bars, 500 nm. (**B**) Representative images of paxillin staining of nLSECs and dLSECs grown on collagen. Nuclei (blue), F-actin (red), Paxillin (green). Scale bars, 5 µm.

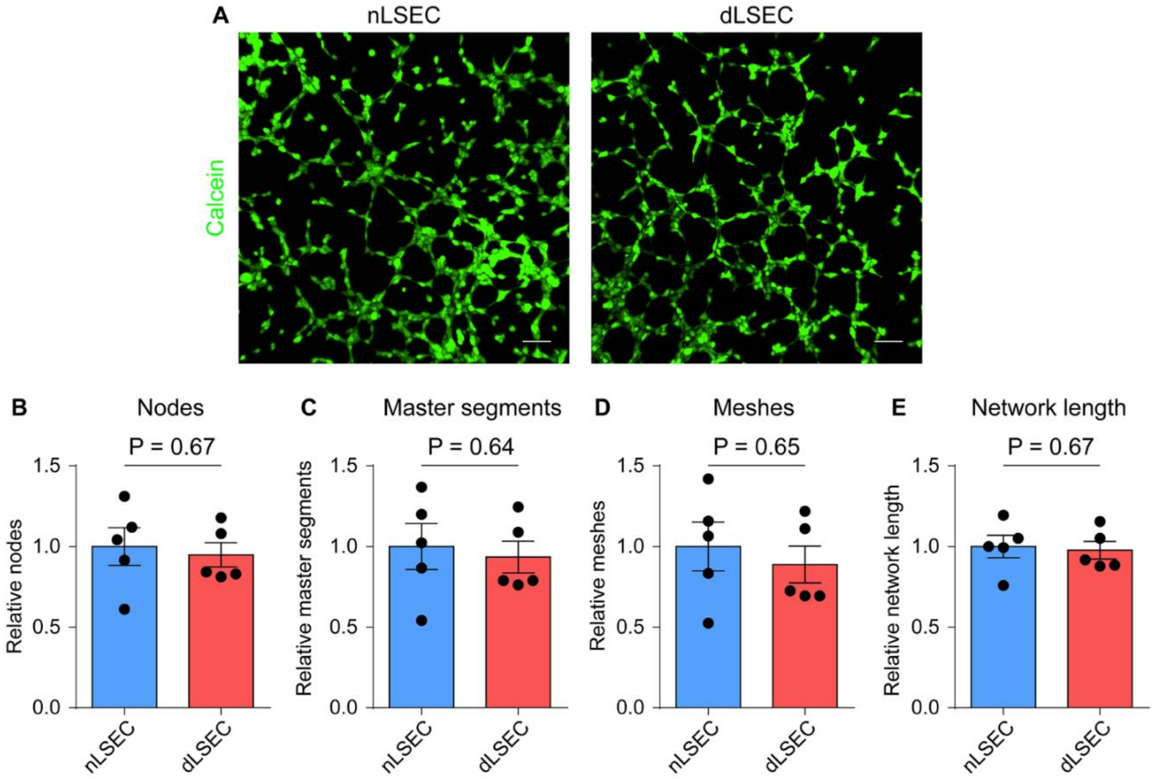

Figure S6. The ability of vascular-endothelial tube formation of dLSECs was similar to that of nLSECs. (**A**) Representative images of calcein staining of nLSECs and dLSECs. Scale bars, 100 µm. (**B** to **E**) Statistical analysis of parameters of tube formation including nodes (**B**), master segments (**C**), meshes (**D**), and network length (**E**) of nLSECs and dLSECs (n = 5, number of pictures analyzed per condition). Statistical analysis was performed using two-tailed unpaired t-test. Results are presented as means ± SEM.

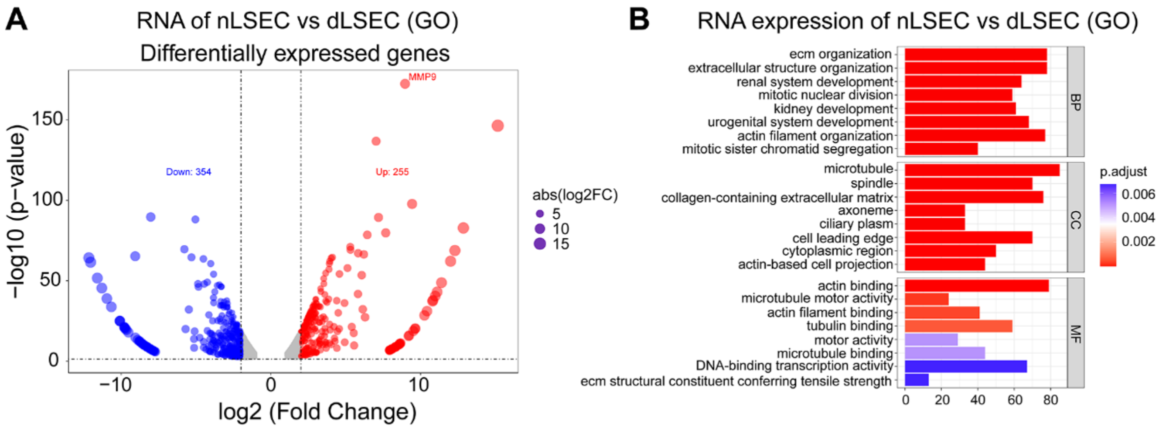

Figure S7. Volcano diagram and gene ontology of differential expression genes in dLSECs compared with nLSECs.

(**A**) Volcano diagram of differential expression genes in dLSECs compared with nLSECs. Analyzed by RNA-seq assays. (**B**) Gene ontology (GO) analysis of significantly enriched genes in dLSECs compared with nLSECs. Analyzed by RNA-seq assays.

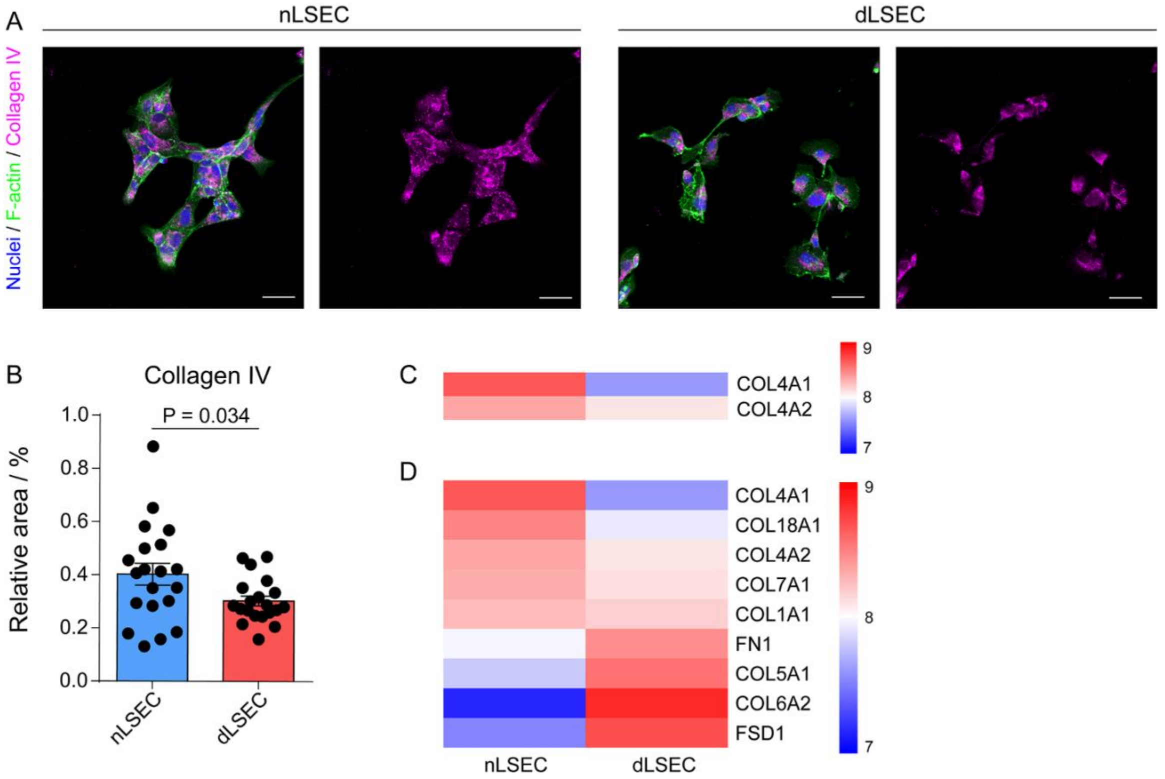

Figure S8. dLSECs showed decreased expression of collagen IV compared with nLSECs. (A) Representative fluorescent images showing the collagen IV staining of nLSECs and dLSECs. Left panel, nuclei (blue), F-actin (green), collagen IV (pink). Right panels, collagen IV (pink). Scale bars, 40 μm. (**B**) Statistical analysis of expression of collagen IV in nLSECs and dLSECs (n = 20, number of pictures analyzed per condition). (**C**) Heatmap view of secreted Collagen IV in supernatants of dLSECs and nLSECs. Analyzed by secretomes of proteomics. (**D**) Heatmap view of secreted ECM-related proteins in supernatants of dLSECs and nLSECs based on logarithmic transformation of secretome counts which is $\log_{10}$ (FPKM). The relative abundance of gene expression was indicated by transition from blue (the lowest), white (middle) and red (the highest). Analyzed by secretomics. Statistical analysis was performed using two-tailed unpaired t-test. Results are presented as means ± SEM.

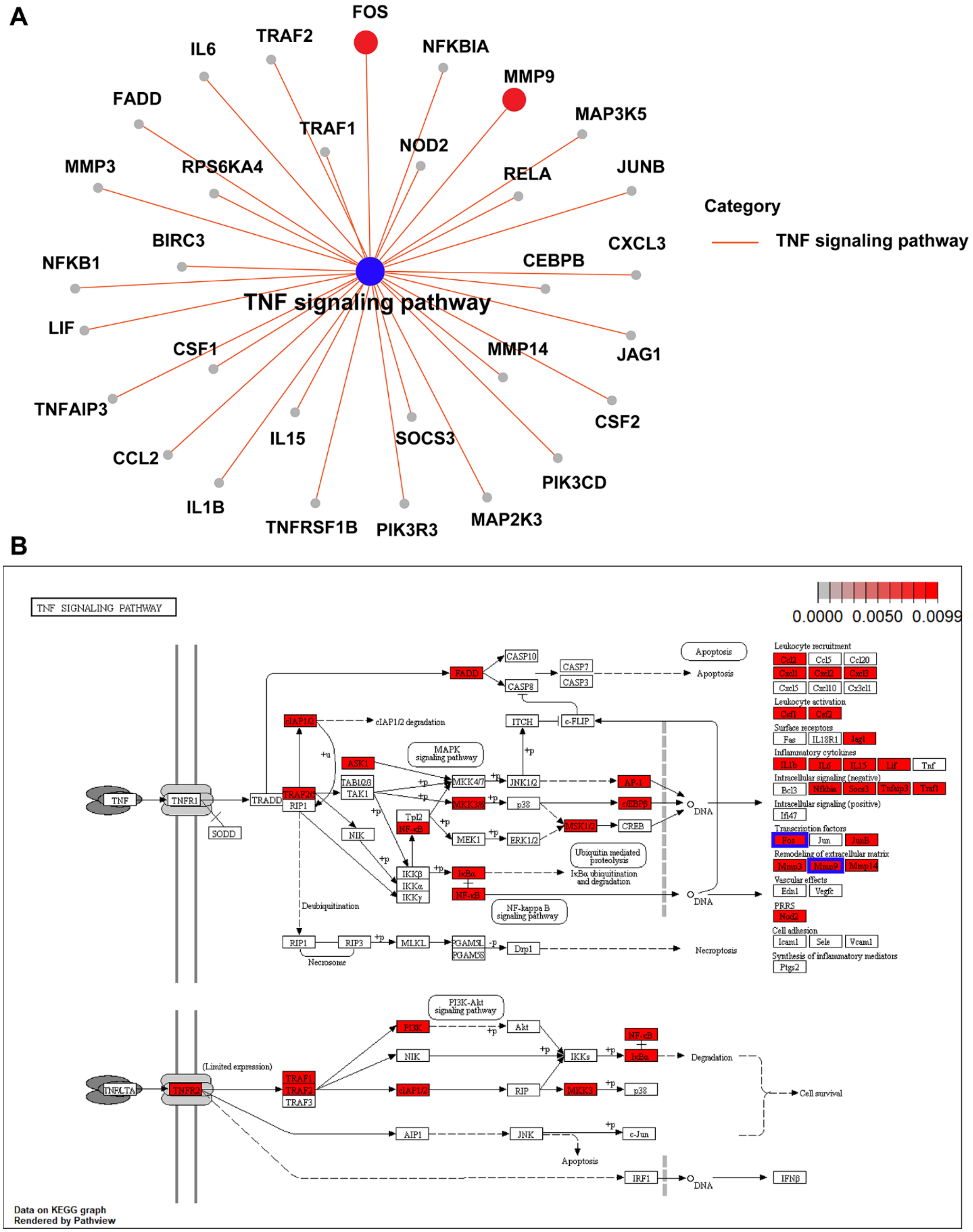

Figure S9. Transcription factor c-Fos acts as a regulator of the TNF signaling pathway, of which multiple pathway members were found to be differentially expressed. (A) Differential genes in TNF signaling pathway. (B) Differential genes enriched in TNF signaling pathway.

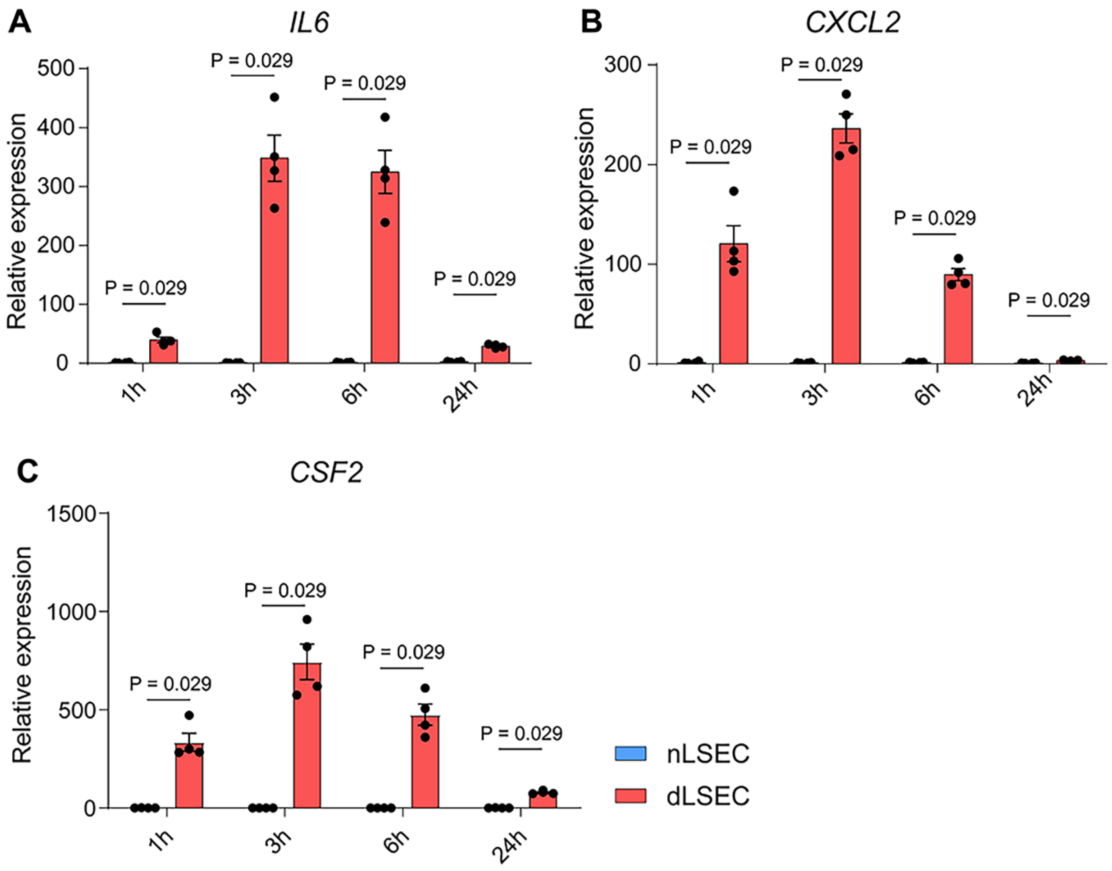

Figure S10. Expression of representative genes regulated by c-Fos in nLSECs and dLSECs. Analyzed by qPCR assay. (A) *IL6,* (B)*CXCL2,* (C)*CSF2*. (n = 4, biological independent samples). Statistical analysis was performed using two-tailed unpaired t-test. Results are presented as means ± SEM.

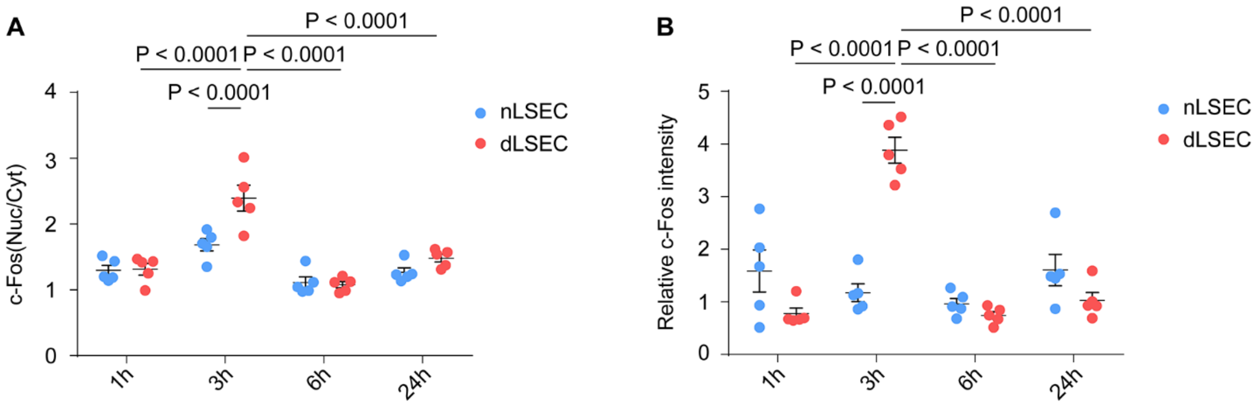

Figure S11. Statistical analysis of c-Fos expression in Figure 5C. (**A**) nuclear/cytoplasm ratio. (**B**) Total expression of c-Fos (n = 5, number of cells analyzed per condition). The statistical analysis was performed using a two-way ANOVA with Turkey test. Results are presented as means ± SEM.

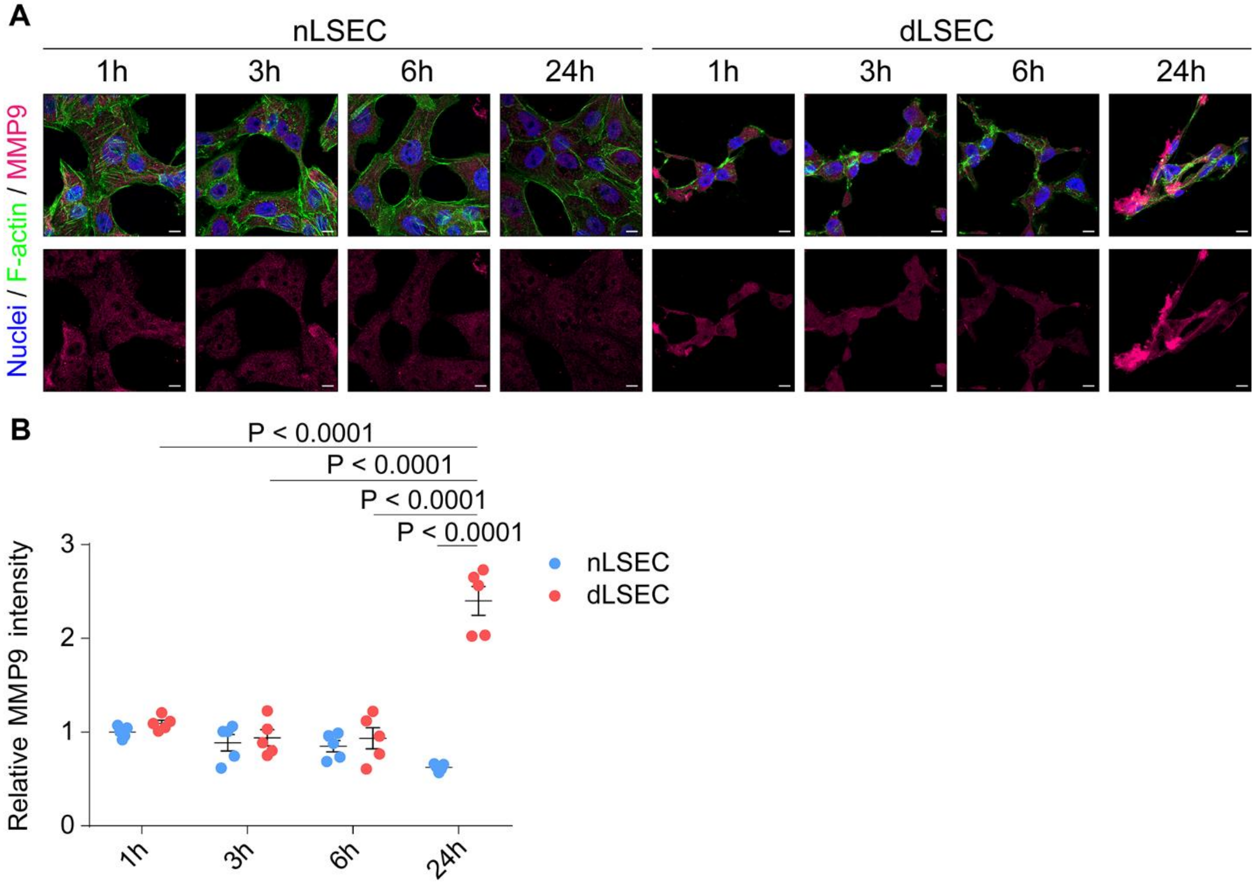

Figure S12. MMP9 expression in dLSECs (related to Figure 5G). (**A**) Representative images of MMP9 staining in nLSECs and dLSECs at different time points after A+P stimulation. Top panel, nuclei (blue), F-actin (green), MMP9 (magenta). Bottom panels, MMP9 (magenta). Scale bars, 10 µm. (**B**) Statistical analysis of relative MMP9 expression in (**A**) (n = 5, number of cells analyzed per condition).

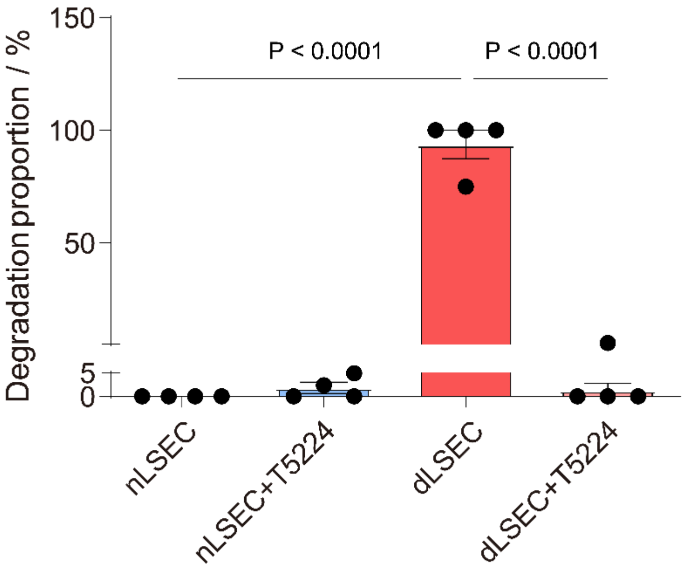

Figure S13. T5224 treatment inhibited ECM-degradation ability of dLSECs. Statistical analysis of collagen degradation mediated by nLSECs and dLSECs with or without treatment of T5224. (n = 4, biological independent samples). The statistical analysis was performed using a one-way ANOVA with Turkey test. Results are presented as means ± SEM.

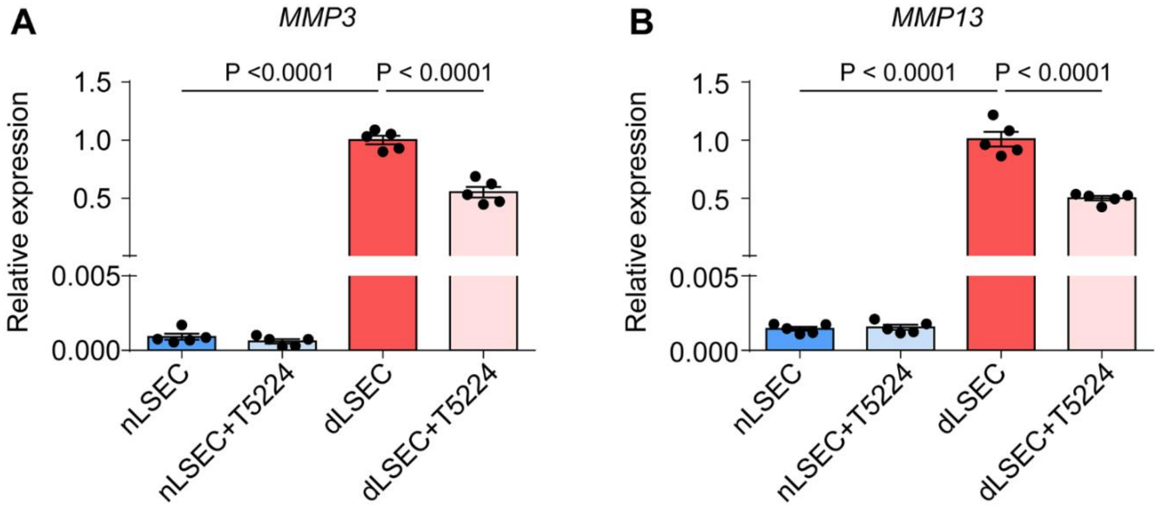

Figure S14. c-Fos regulated mRNA expression of *MMP3, MMP13* in dLSECs. (**A** and **B**) Relative mRNA expression of *MMP3* (**A**)*, MMP13* (**B**) in nLSECs and dLSECs with or without the treatment of T5224 (n = 5, number of cells analyzed per condition). The statistical analysis was performed using a one-way ANOVA with Turkey test. Results are presented as means ± SEM.

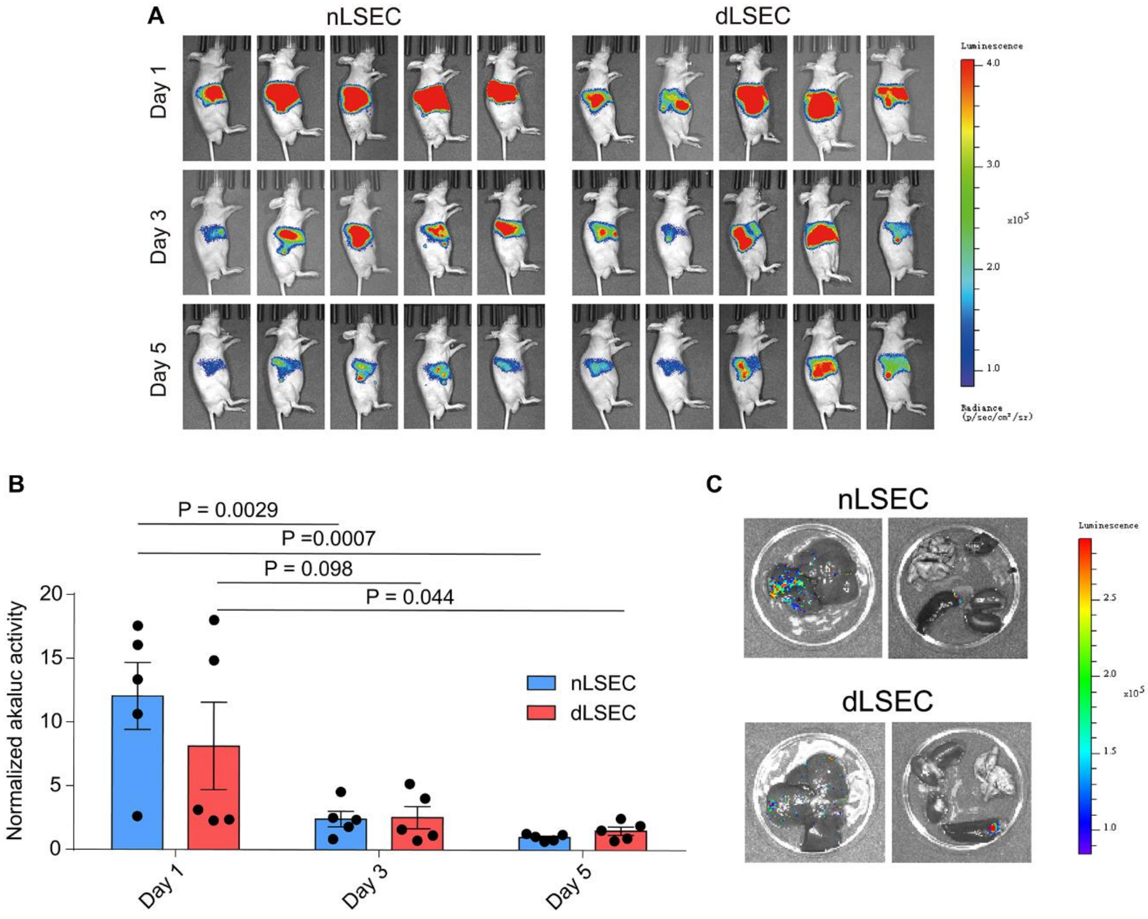

Figure S15. nLSECs and dLSECs resided in the liver upon one-dose intrasplenic injection at day 5 post treatment *in vivo*. (**A**) *In vivo* bioluminescent imaging of nLSECs and dLSECs resided in mice at day 1, 3, and 5 post injection. (**B**) Statistical analysis of bioluminescence signals *in vivo* at day 1, 3, and 5 post injection (n = 5, biologically independent mice per group). (**C**) Representative bioluminescent imaging of cells in livers and other organs at day 5 post treatment.

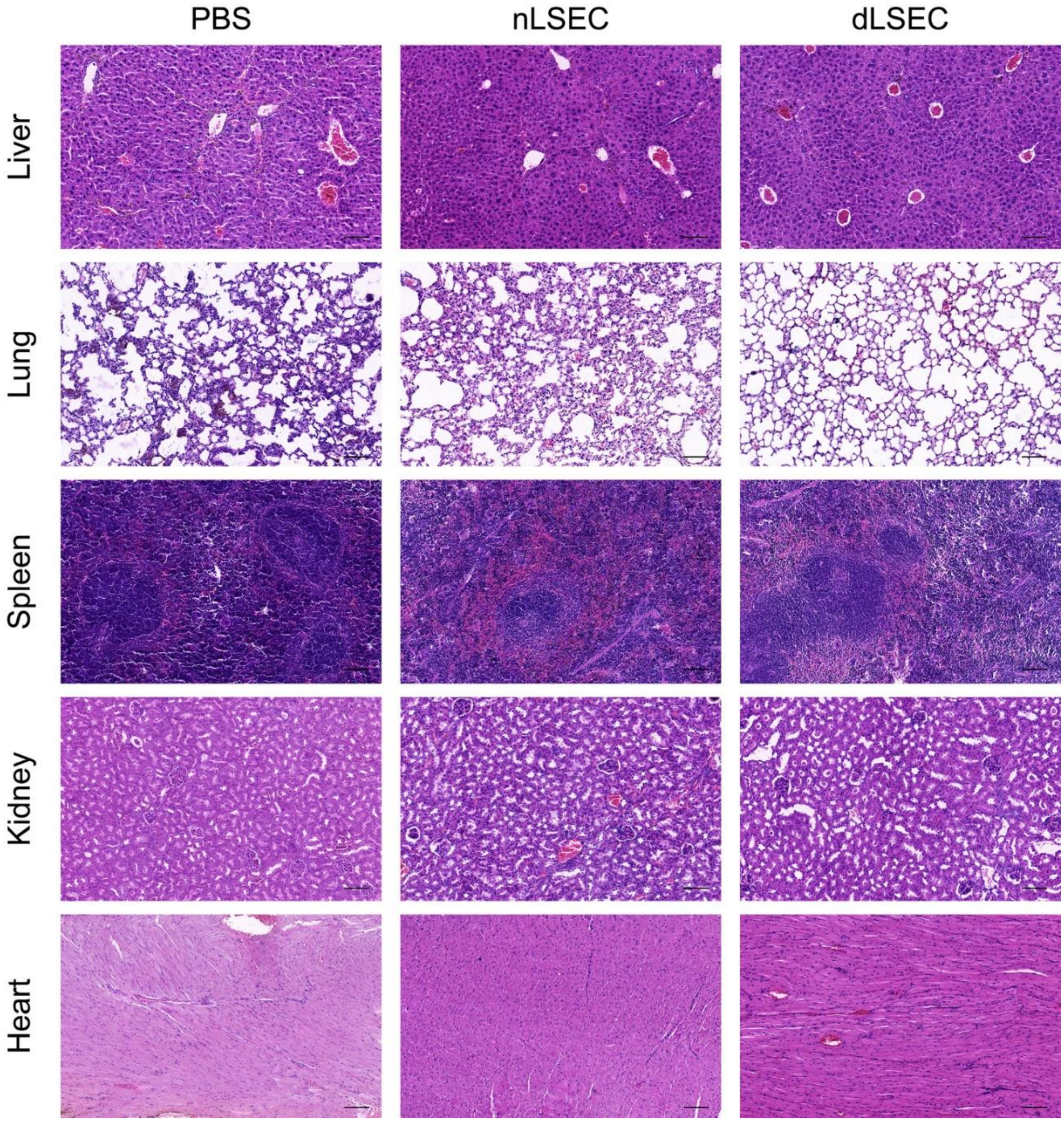

Figure S16. Intrasplenic administration of dLSEC treatment did not cause histological abnormity in the major organs in CCl_4-induced liver fibrosis model. Representative HE images of livers, lungs, spleens, kidneys and hearts from mice treated by PBS, nLSECs and dLSECs. Scale bars, 100 µm.

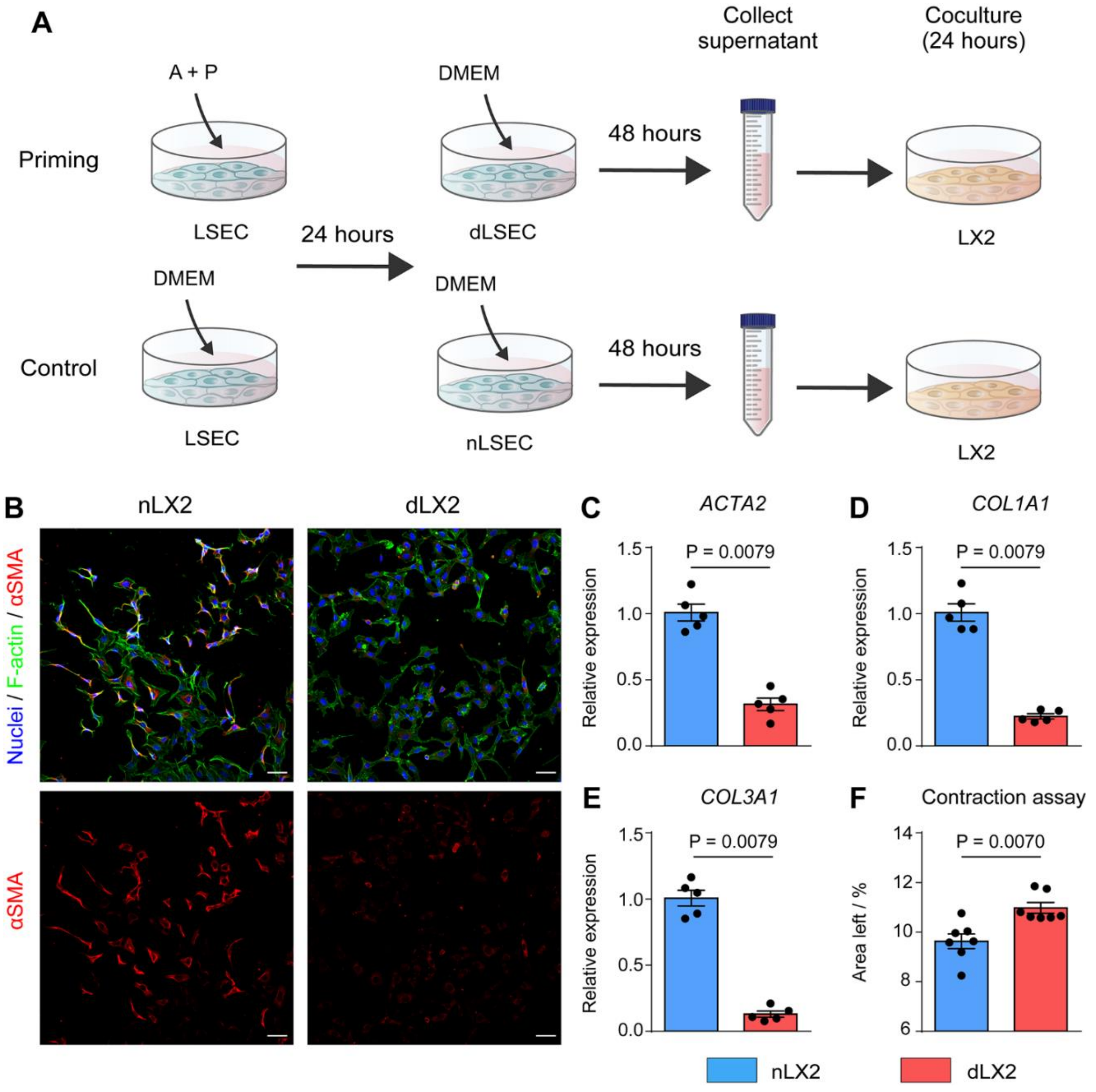

Figure S17. Secretome of dLSECs could deactivate LX-2 *in vitro*. (**A**) Schematic of treating LX2 with conditioned medium from nLSECs and dLSECs. (**B**) Representative images of αSMA staining of nLX2s and dLX2s. Top panel, nuclei (blue), F-actin (green), αSMA (red). Bottom panels, αSMA (red). Scale bars, 50 μm. (**C**) (**D**) (**E**) Relative mRNA expression of *ACTA2* (**C**), *COL1A1* (**D**), *COL3A1* (**E**) in nLX2s and dLX2s. (**F**) Statistical analysis of LX-2 induced collagen matrix contraction (n = 7, biologically independent samples per group). Statistical analysis was performed using two-tailed unpaired t-test. Results are presented as means ± SEM.

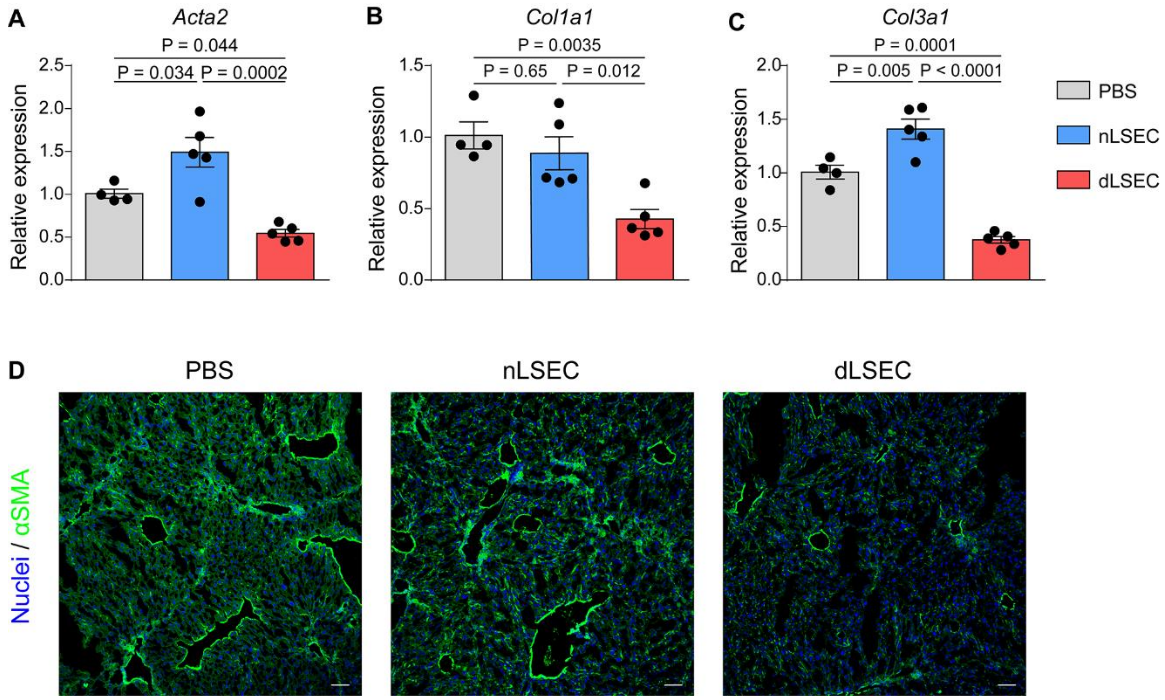

Figure S18. Intrasplenic administration of dLSECs could deactivate HSCs *in vivo*. (**A** to **C**) Relative mRNA expression of *Acta2, Col1a1 and Col3a1* in liver tissues treated by PBS, nLSECs and dLSECs (n ≥ 4, biologically independent mice per group). (**D**) Representative images of αSMA stained by immunofluorescence in liver tissues treated by PBS, nLSECs and dLSECs. Scale bars, 100 µm. The statistical analysis was performed using a one-way ANOVA with Turkey test. Results are presented as means ± SEM.

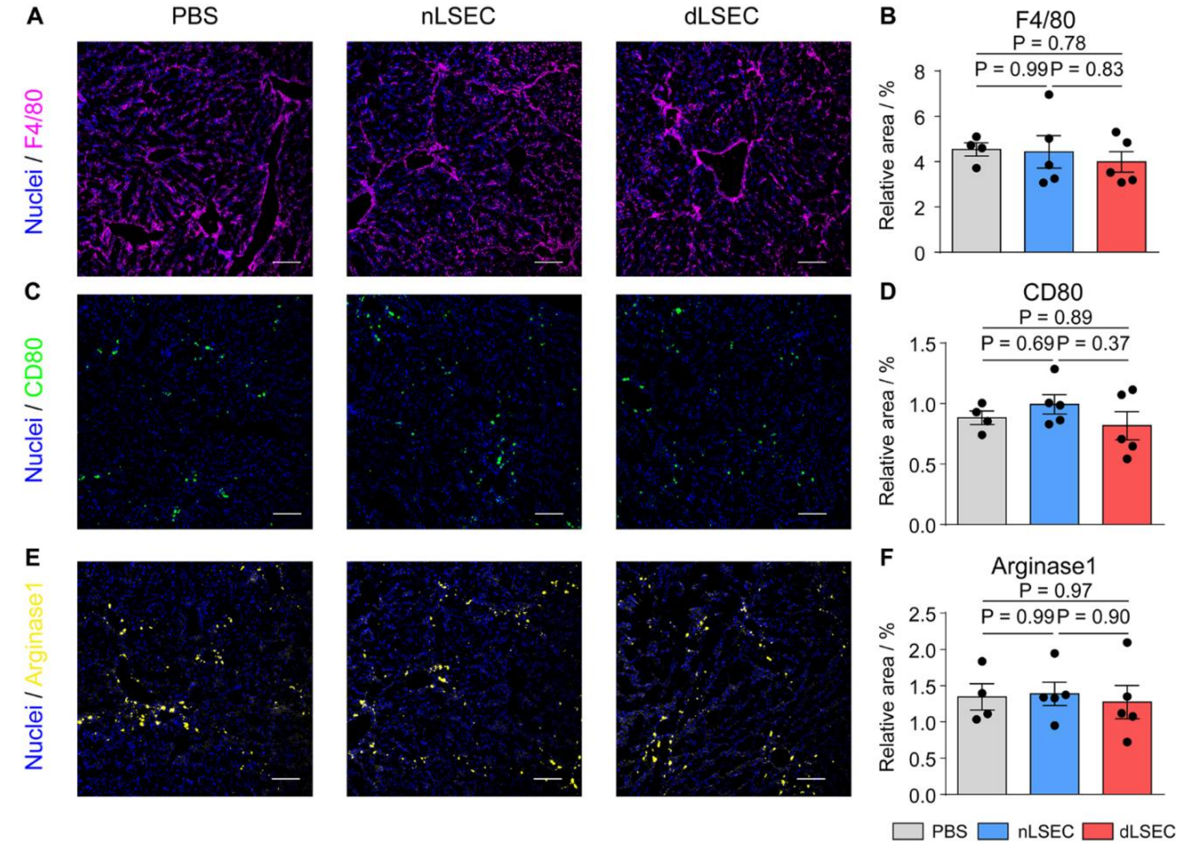

Figure S19. Intrasplenic administration of dLSECs did not induce an increase in the expression of F4/80, CD80 and Arginase-1 *in vivo*. (**A to F**) Representative immunofluorescent images and statistical analysis of expression of F4/80, CD80 and Arginase-1 in liver tissues treated by PBS, nLSECs and dLSECs (n ≥ 4, biologically independent mice per group). Scale bars, 150 µm. The statistical analysis was performed using a one-way ANOVA with Turkey test. Results are presented as means ± SEM.

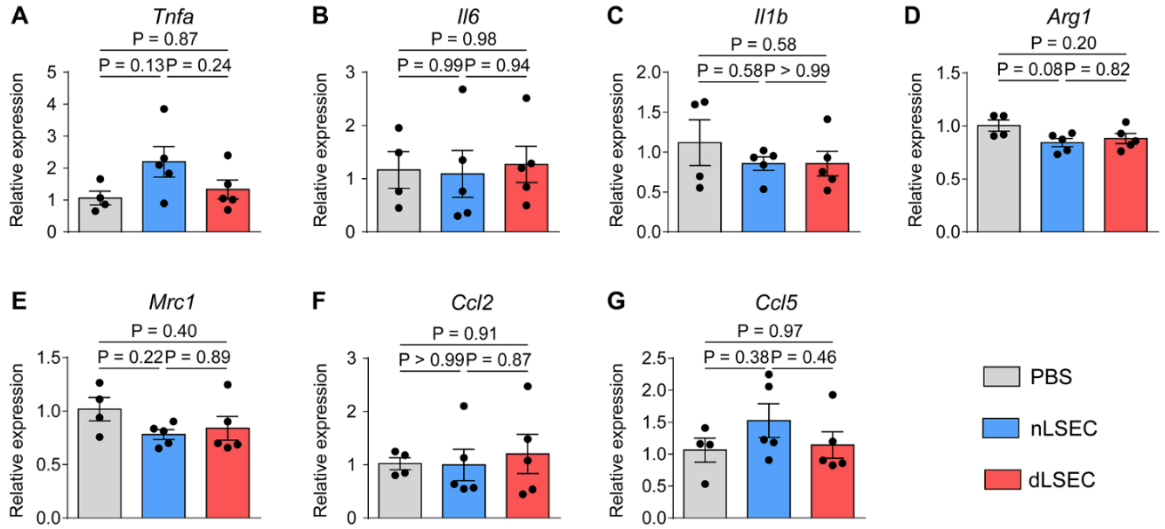

Figure S20. Intrasplenic administration of dLSECs did not increase the expression of inflammation-related genes *in vivo*. (**A to G**) Relative mRNA expression of *Tnfa, Il6, Il1b, Arg1, Mrc1, Ccl2, Ccl5* in liver tissues treated by PBS, nLSECs and dLSECs (n ≥ 4, biologically independent mice per group). The statistical analysis was performed using a one-way ANOVA with Turkey test. Results are presented as means ± SEM.

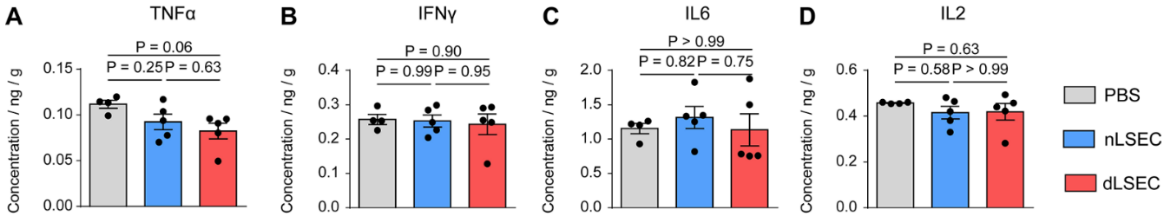

Figure S21. Intrasplenic administration of dLSECs did not increase the expression of pro-inflammatory cytokines in liver homogenates *in vivo*. (**A to D**) Pro-inflammatory cytokines expression of TNF, IFNγ, IL6, IL2 in liver homogenate treated by PBS, nLSECs and dLSECs (n ≥ 4, biologically independent mice per group). The statistical analysis was performed using a one-way ANOVA with Turkey test. Results are presented as means ± SEM.

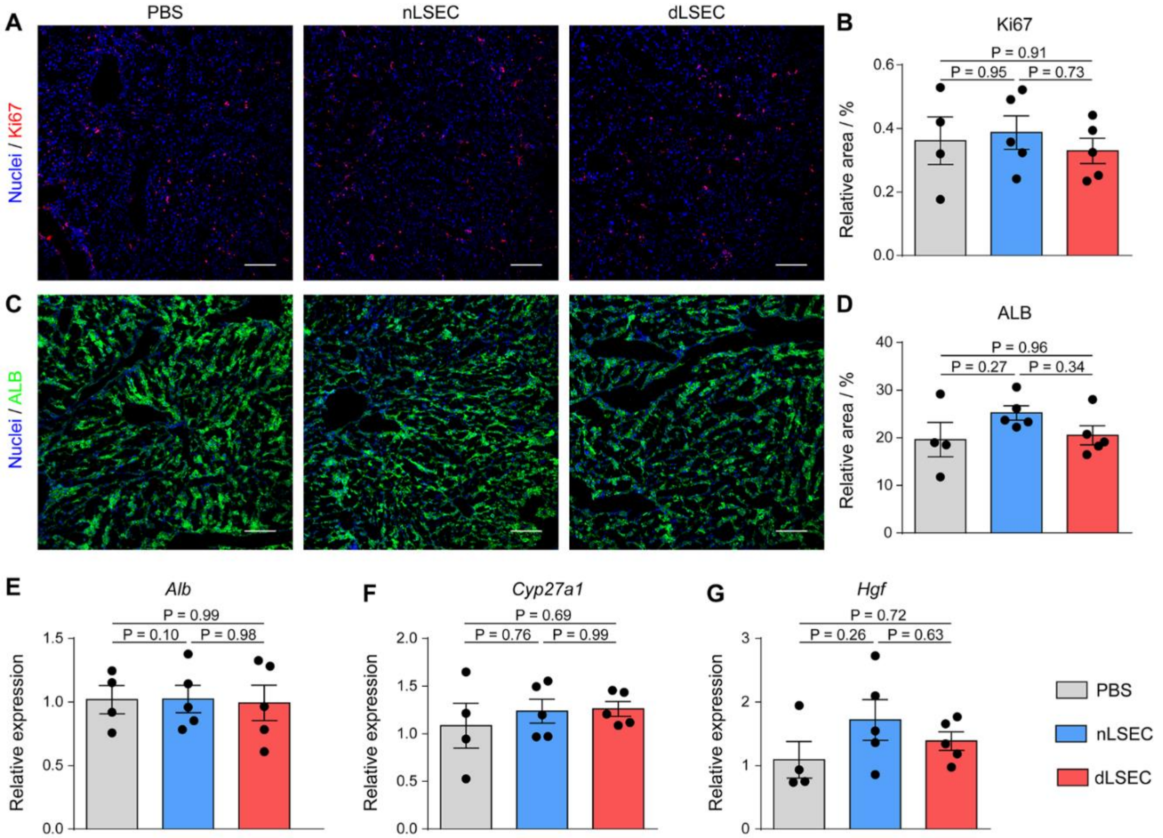

Figure S22. Intrasplenic administration of dLSECs did not induce liver regeneration *in vivo*. (**A to D**) Representative immunofluorescent images and statistical analysis of expression of Ki67 and ALB in liver tissues treated by PBS, nLSECs and dLSECs (n ≥ 4, biologically independent mice per group). Scale bars, 150 μm. (**E to G**) Relative mRNA expression of *Alb, Cyp27a1 and Hgf* in liver tissues treated by PBS, nLSECs and dLSECs (n ≥ 4, biologically independent mice per group). The statistical analysis was performed using a one-way ANOVA with Turkey test. Results are presented as means ± SEM.

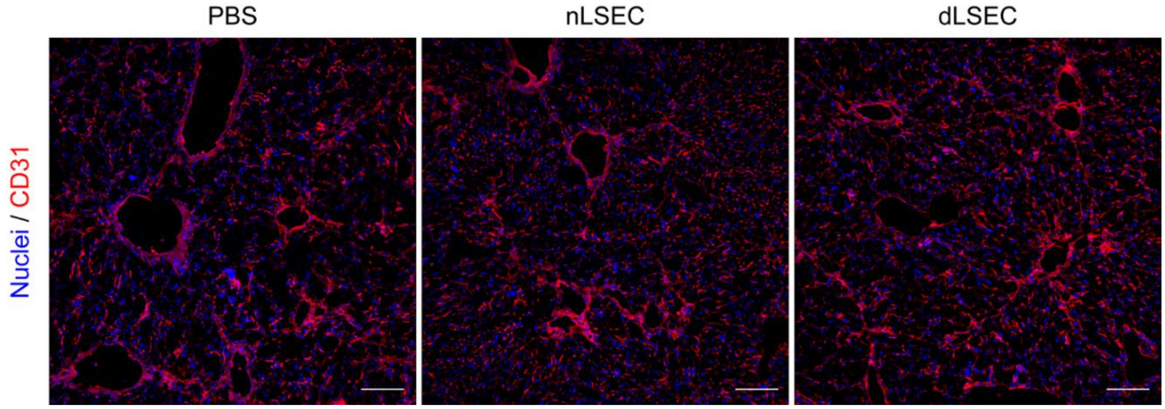

Figure S23. Intrasplenic administration of dLSECs did not increase the expression of CD31 *in vivo*. Representative images of CD31 stained by immunofluorescence in liver tissues treated by PBS, nLSECs and dLSECs. Scale bars, 150 μm.

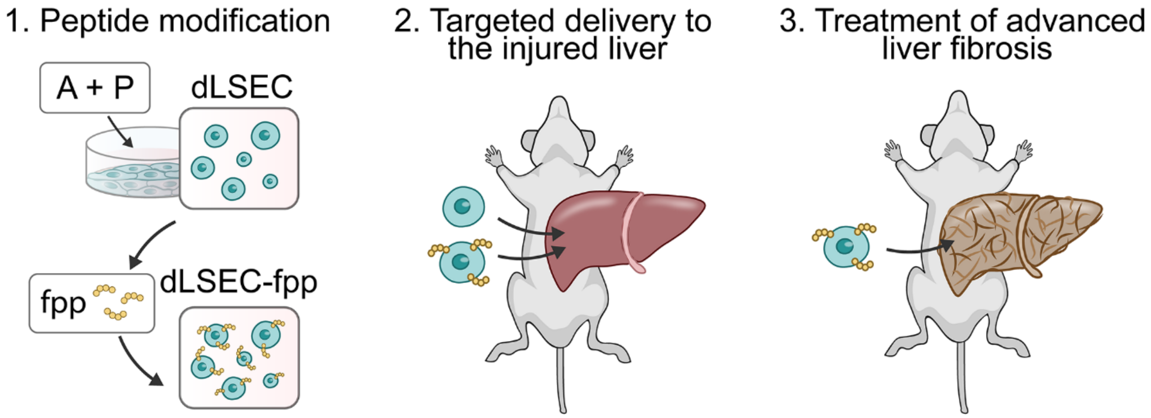

Figure S24. Schematic of constructing liver-targeting dLSECs by membrane modification of liver-targeting functional peptide, fpp. The liver targeting ability of constructed cells (i.e. dLSEC-fpp and nLSEC-fpp) was verified in a liver injury model and the fibrosis treatment was performed in a CCl_4-induced advanced liver fibrosis model.

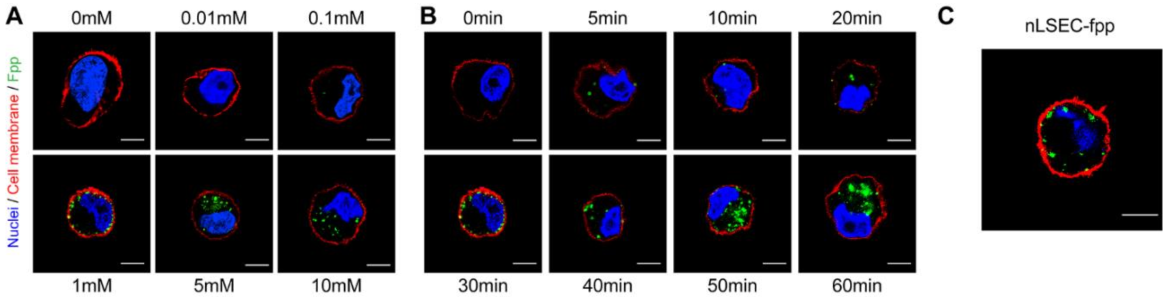

Figure S25. Constructing dLSEC-fpp by membrane modification of liver-targeting peptide. (**A**) Representative images of FITC-labeled liver-targeting peptide, fpp, modified on dLSECs treated by different fpp concentrations for a modification time of 30 min. Nuclei (blue), fpp (green), cell membrane (red). Scale bars, 10 μm. (**B**) Representative images of fpp modified on dLSECs treated for different time at a fpp concentration of 1mM. Nuclei (blue), fpp (green), cell membrane (red). Scale bars, 10 μm. (**C**) Representative images of fpp modified on nLSECs. Modification time: 30 min. Modification concentration: 1mM. Nuclei (blue), fpp (green), cell membrane (red). Scale bars, 10 μm.

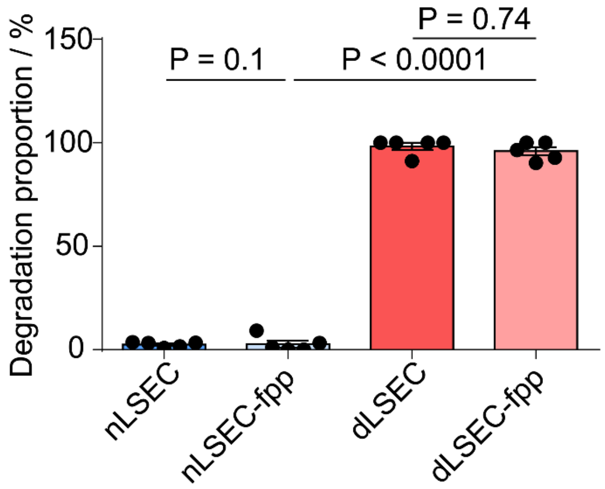

Figure S26. dLSEC-fpp showed high ECM degradation ability comparable to the unmodified dLSECs. *In vitro* characterization of collagen degradation ability of nLSECs, nLSEC-fpp, dLSECs and dLSEC-fpp (n = 5, biological independent samples). The statistical analysis was performed using a one-way ANOVA with Turkey test. Results are presented as means ± SEM.

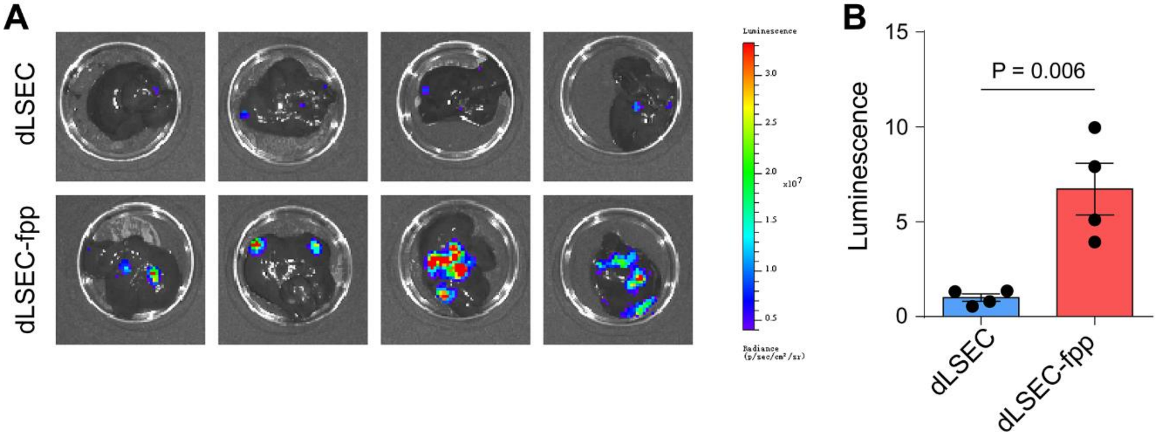

Figure S27. dLSEC-fpp could target the damaged liver. (**A**) Bioluminescent imaging of dLSECs and dLSEC-fpp in treated livers after 3 days post administration in a liver injury model. (**B**) Statistical analysis of bioluminescence signals in (**A**) (n = 4, biologically independent mice per group). The statistical analysis was performed using two-tailed unpaired t-test. Results are presented as means ± SEM.

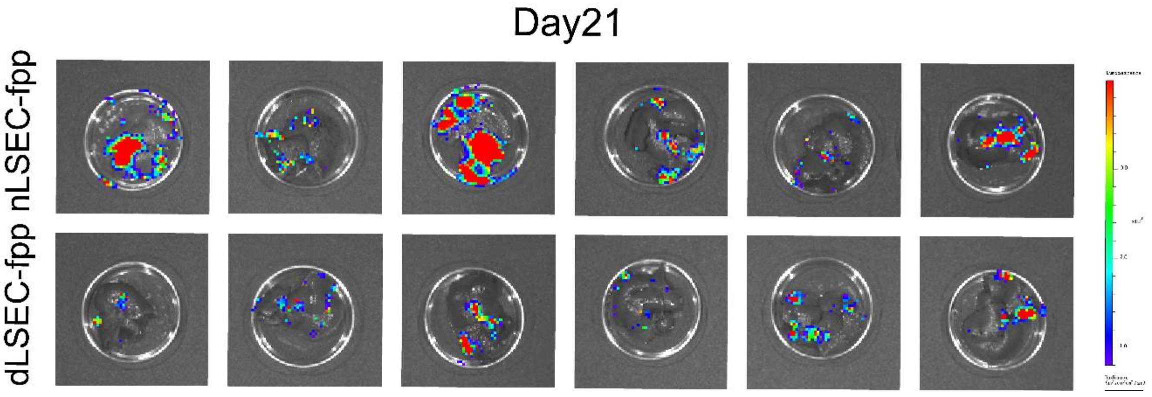

Figure S28. nLSEC-fpp and dLSEC-fpp resided in the liver upon systemic injection at day 21 post treatment *in vivo*. Bioluminescent imaging of nLSEC-fpp and dLSEC-fpp in treated livers after the final administration.

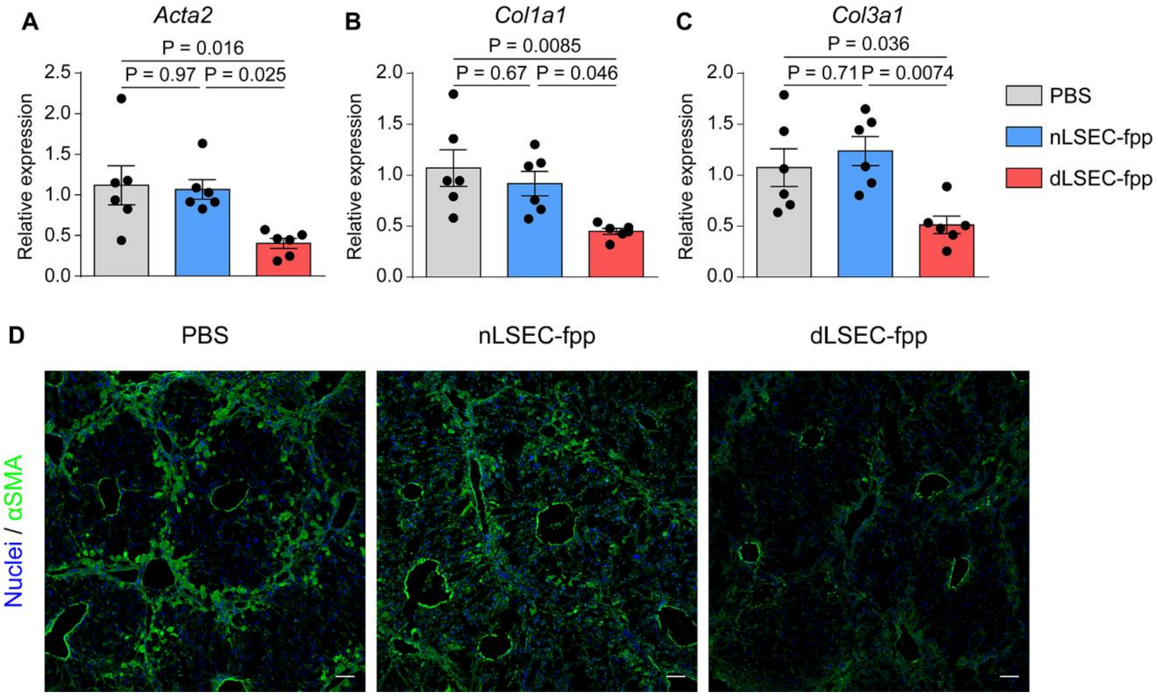

Figure S29. Systemic administration of dLSEC-fpp could deactivate HSCs *in vivo.* (**A**, to **C**) Relative mRNA expression of *Acta2, Col1a1 and Col3a1* in liver tissues treated by PBS, nLSEC-fpp and dLSEC-fpp (n = 6, biologically independent mice per group). (**D**) Representative images of αSMA stained by immunofluorescence in liver tissues treated by PBS, nLSEC-fpp and dLSEC-fpp. Scale bars, 100 μm. The statistical analysis was performed using a one-way ANOVA with Turkey test. Results are presented as means ± SEM.

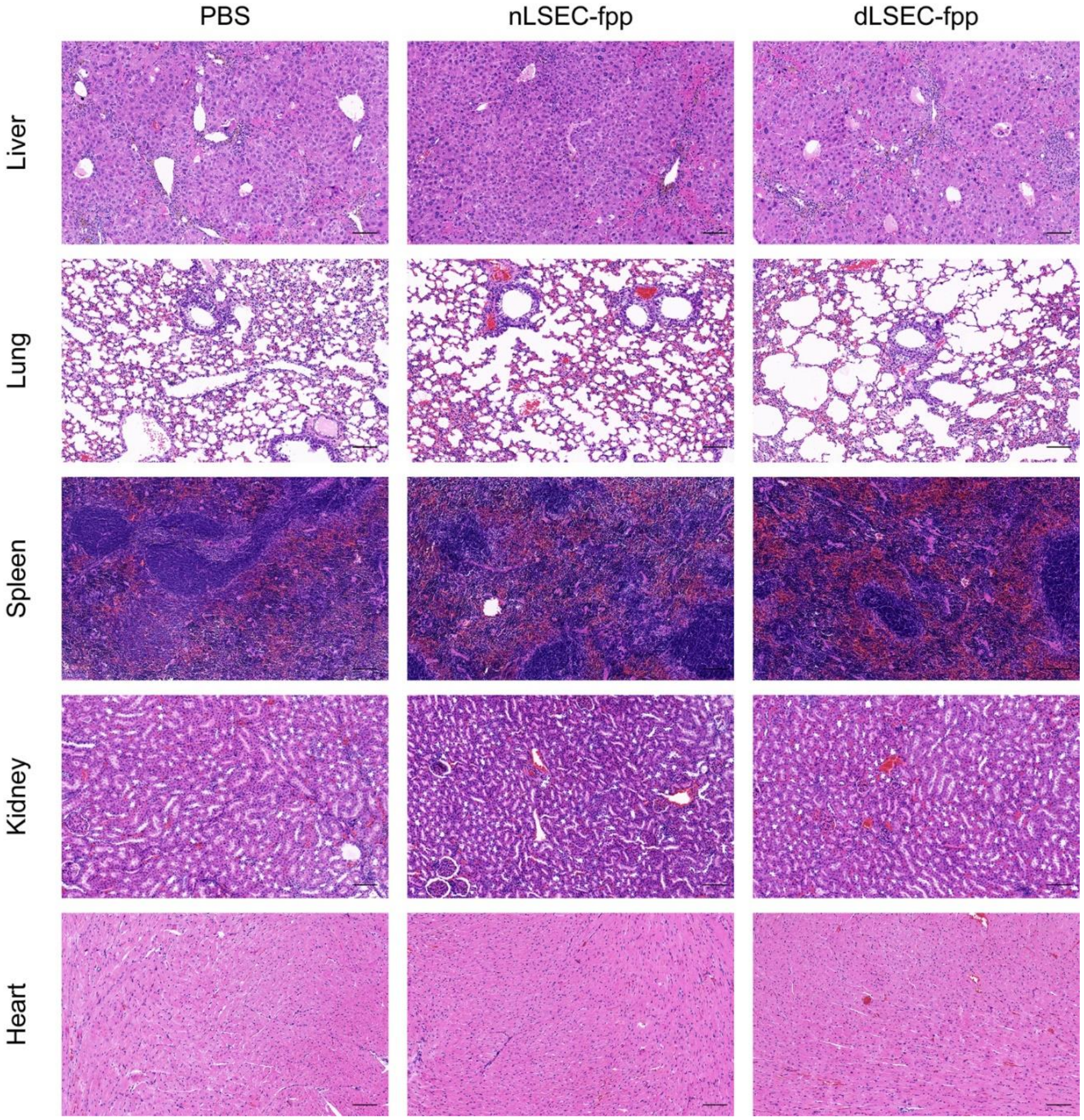

Figure S30. Systemic administration of dLSEC-fpp did not cause histological abnormity in the major organs in CCl_4-induced liver fibrosis model. Representative HE images of livers, lungs, spleens, kidneys, hearts from mice treated by PBS, nLSEC-fpp and dLSEC-fpp. Scale bars, 100 μm.

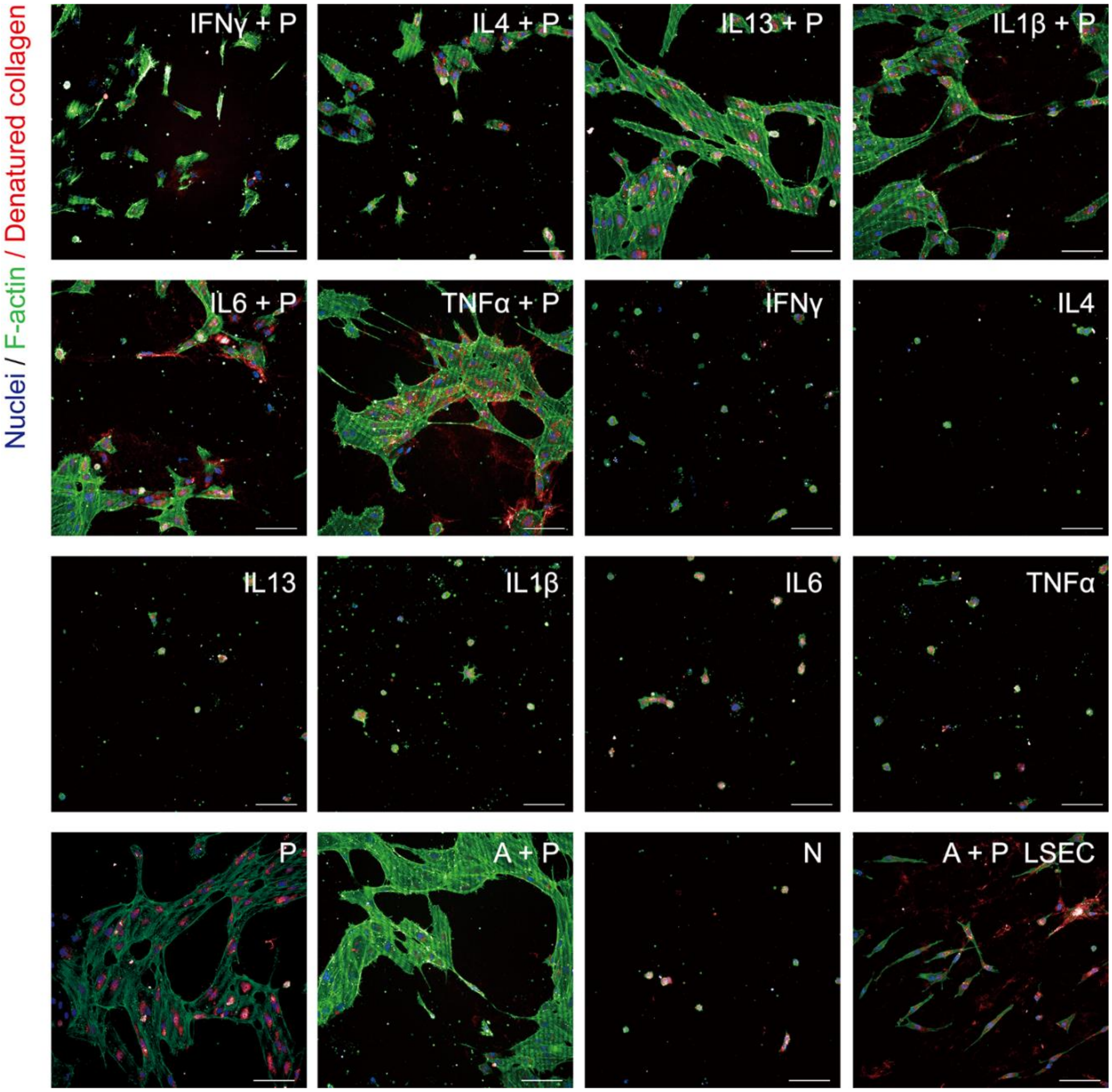

Figure S31. High-content fluorescent images validated the highly ECM degradation ability of dHUVECs. Representative high-content fluorescent images of collagen matrix being degraded by HUVECs stained by CHP assay. Nuclei (blue), F-actin (green), denatured collagen (red). Scar bar, 100μm.

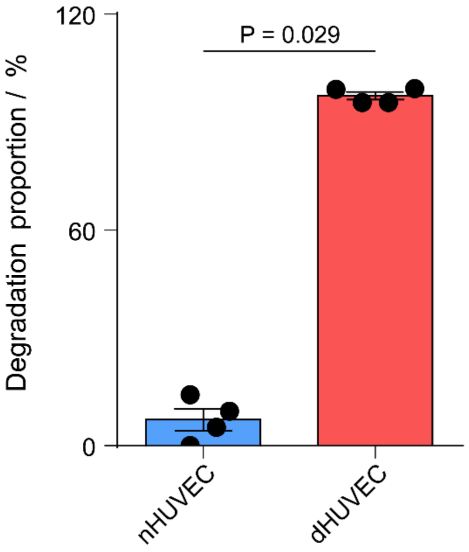

Figure S32. Highly ECM degradation ability of dHUVECs was validated by CEDSS. The collagen matrix degradation abilities of nHUVECs and dHUVECs are characterized by CEDSS (n =4, biological independent samples). Statistical analysis was performed using two-tailed unpaired t-test. Results are presented as means ± SEM.

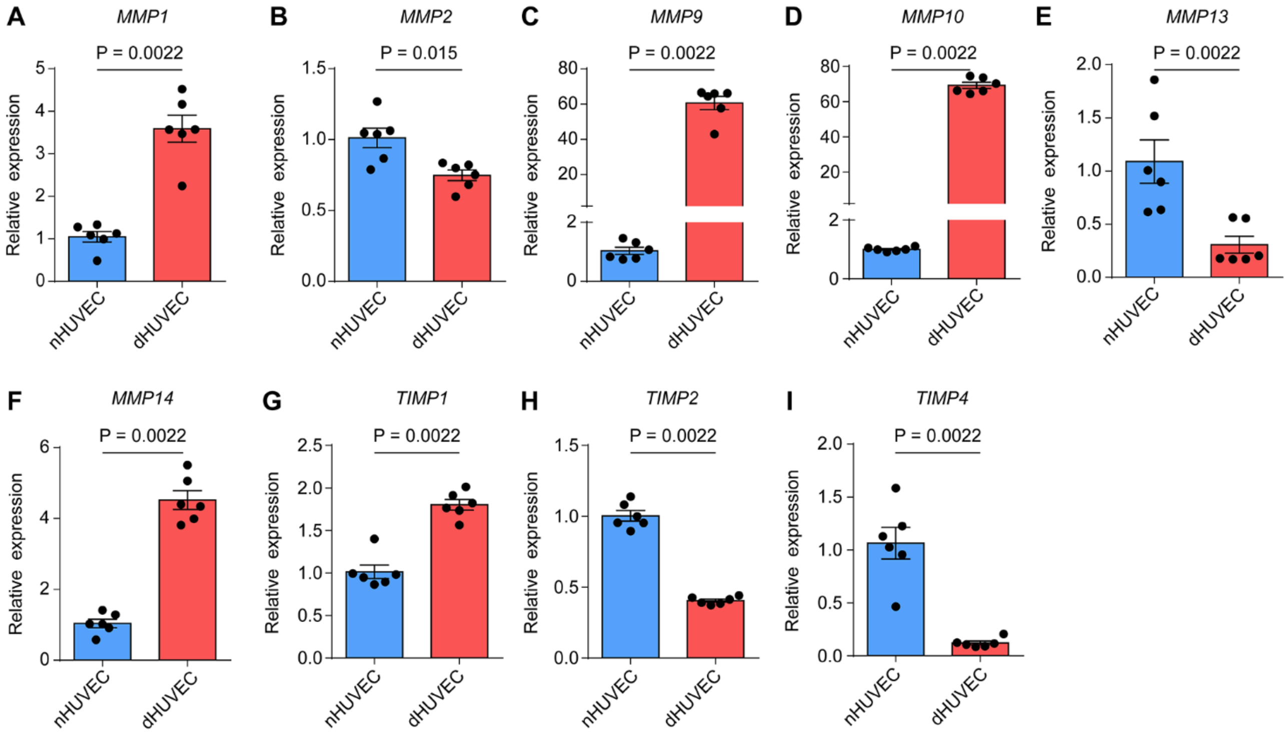

Figure S33. dHUVECs showed high expression of MMPs genes. Relative mRNA expression of *MMP1, MMP2, MMP9, MMP10, MMP13, MMP14, TIMP1, TIMP2, TIMP4* in nHUVECs and dHUVECs. (n = 6, biological independent samples). Statistical analysis was performed using two-tailed unpaired t-test. Results are presented as means ± SEM.

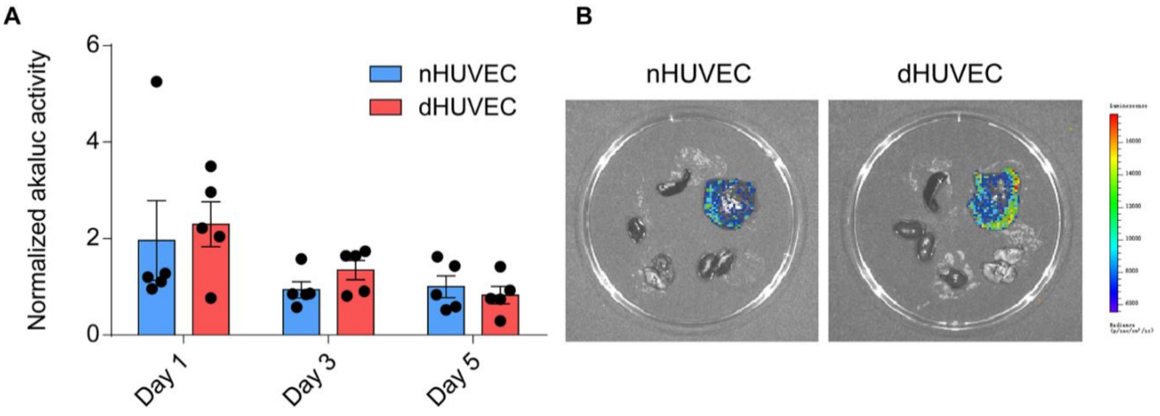

Figure S34. Intrasplenic administration of dHUVECs and nHUVECs could reside in livers after 5 days post administration. (**A**) Statistical analysis of bioluminescence signals *in vivo* at day 1, 3, and 5 post administration (n = 5, biologically independent mice per group). (**B**) In vivo bioluminescent imaging of livers and other organs treated by PBS, nHUVECs and dHUVECs resided in mice on day 5 post administration.

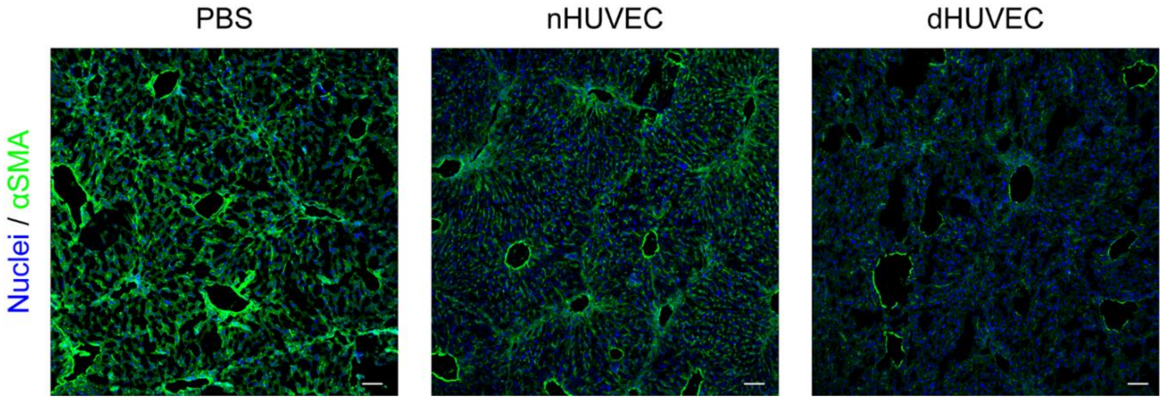

Figure S35. Intrasplenic administration of dHUVECs could deactivate HSCs *in vivo*. Representative images of αSMA stained by immunofluorescence in liver tissues treated by PBS, nHUVECs and dHUVECs. Scale bars, 100 μm.

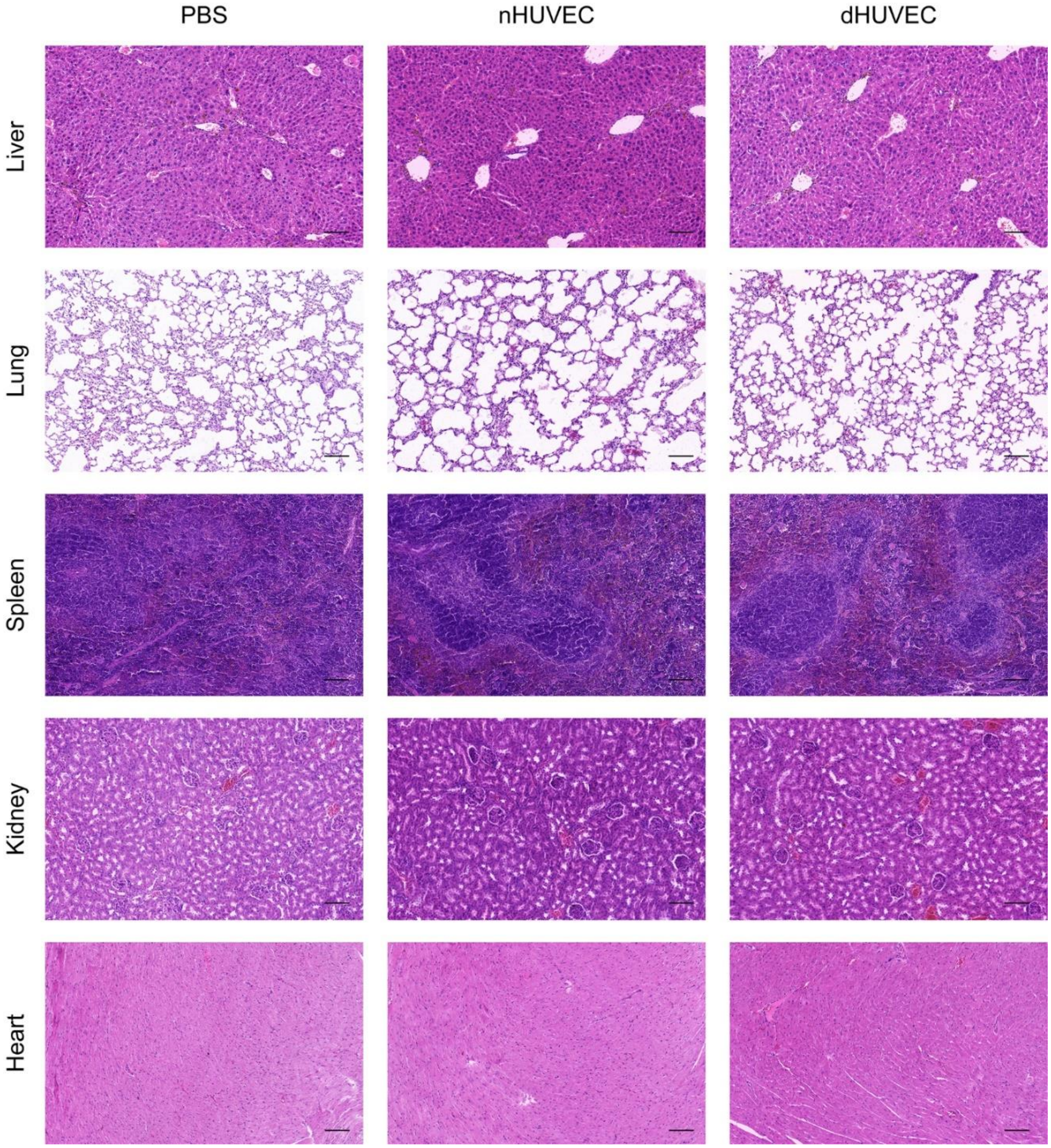

Figure S36. Intrasplenic administration of dHUVECs did not cause histological abnormity in the major organs in CCl_4-induced liver fibrosis model. Representative HE images of livers, lungs, spleens, kidneys and hearts from mice treated by PBS, nHUVECs and dHUVECs. Scale bars, 100 μm.

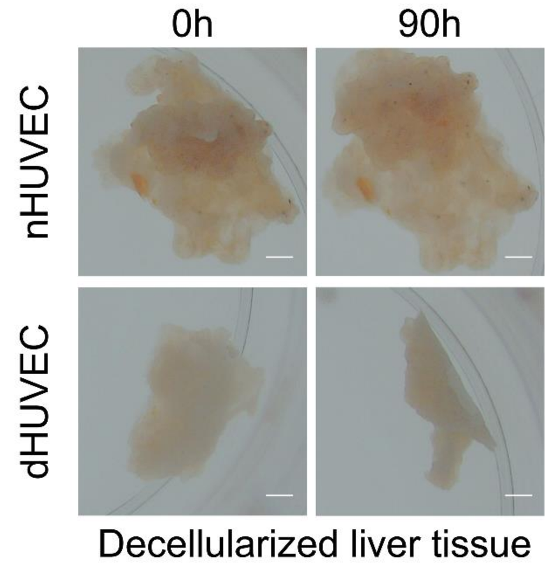

Figure S37. dHUVECs could also degrade human decellularized cirrhotic liver tissue. Representative images of human decellularized cirrhotic liver tissue degraded by nHUVECs and dHUVECs. Scale bars, 0.5mm.

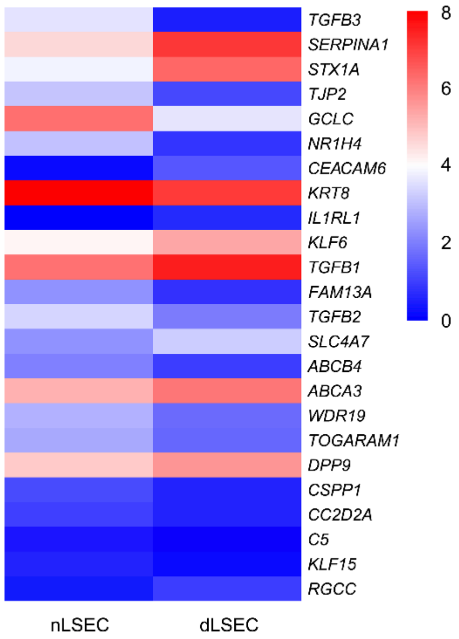

Figure S38. Heapmap view of the expression of representative genes related liver fibrosis. Analyzed by RNA-seq assays. Relative expression of genes was determined based on logarithmic transformation of FPKM counts which is $\log_{10}$ (FPKM+1). The relative abundance of gene expression was indicated by transition from blue (the lowest), white (middle) and red (the highest).

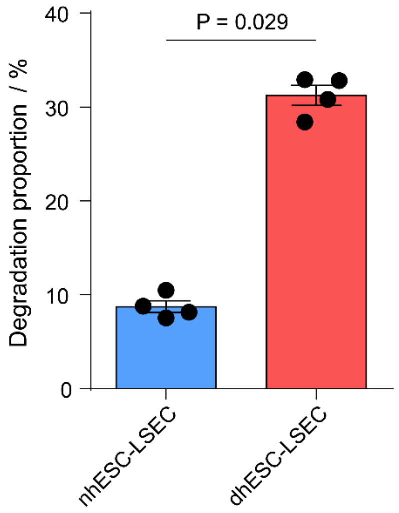

Figure S39. dhESC-LSECs could also degrade collagen upon stimulation by A and P. Statistical analysis of collagen degradation mediated by nhESC-LSECs and dhESC-LSECs (n = 4, biological independent samples). Statistical analysis was performed using two-tailed unpaired t-test. Results are presented as means ± SEM.

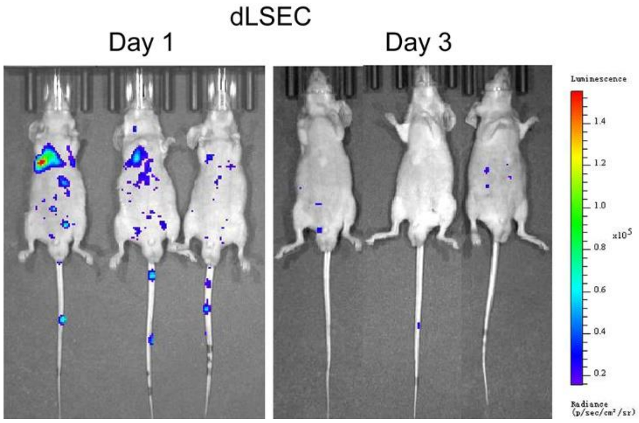

Figure S40. dLSEC administrated by intravenous injection could not target the liver effectively. Bioluminescent imaging of dLSECs resided in mice at day 1 and 3 post injection.

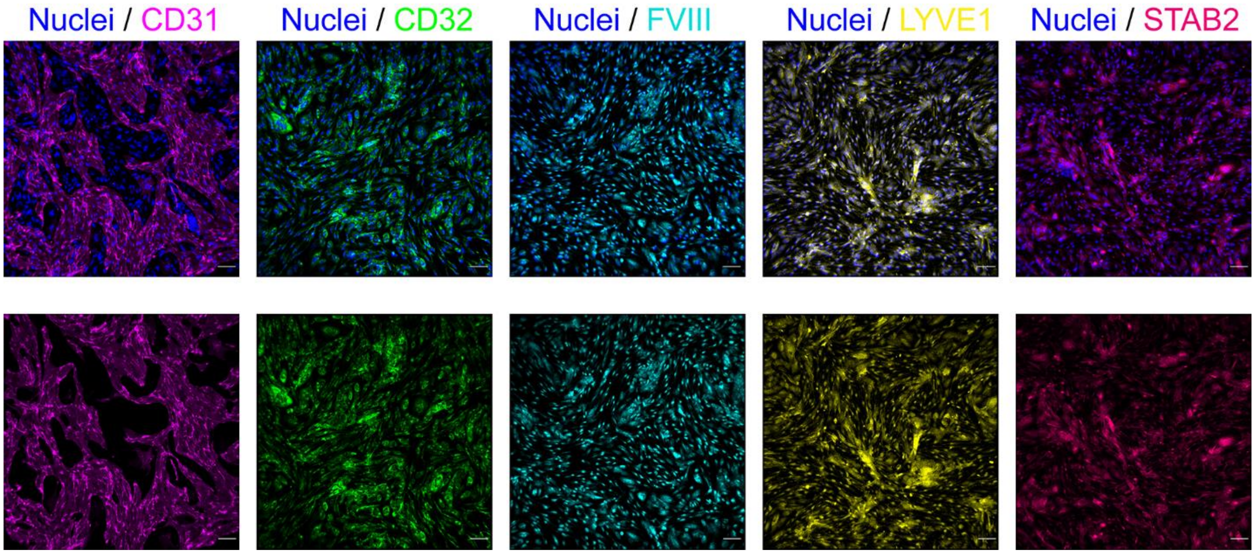

Figure S41. Characterization of successful construction of hESC-LSECs. Representative immunofluorescent images of staining of typical LSECs marker in hESC-LSECs, including CD31, CD32, FVIII, LYVE1 and STAB2**.** Scale bars, 100 μm.

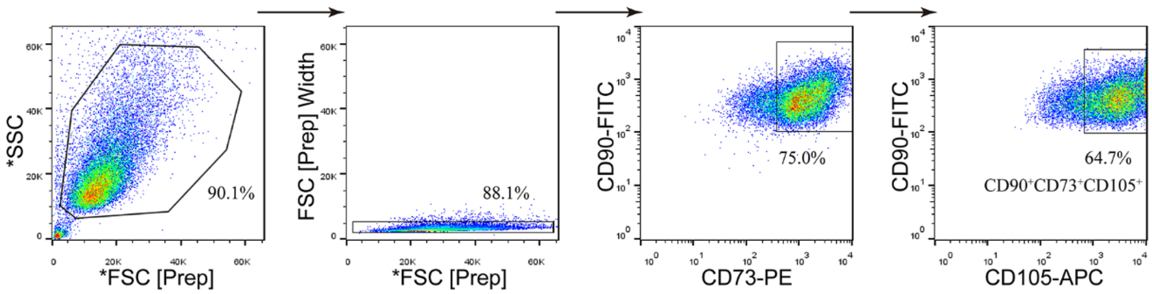

Figure S42. Fluorescence activated Cell Sorting (FACS) of hESC-MSCs. The constructed hESC-MSCs were sorted according to the expression of typical MSCs' markers. CD90+CD73+CD105+ triple-positive cell population was sorted with high yield up to 64.7%.

Table S1. Information of cell candidates screened by CEDSS.

HL60	HL60<br>P	HL60<br>A	HL60<br>A+P	HL60<br>fMLP	HL60<br>DMSO	HL60<br>fMLP<br>+DMSO	LX2	LX2<br>P	LX2<br>A
LX2<br>A+P	LX2<br>TGFβ	LSEC(FBS)	LSEC(FBS)<br>P	LSEC(FBS)<br>A	LSEC(FBS)<br>A+P	ADMSC	ADMSC<br>P	ADMSC<br>A	ADMSC<br>A+P
ESC-LSEC	ESC-LSEC<br>P	ESC-LSEC<br>A	ESC-LSEC<br>A+P	Raw264.7	Raw264.7<br>IL4	Raw264.7<br>IL13	Raw264.7<br>LPS	Raw264.7<br>P	Raw264.7<br>A
Raw264.7<br>A+P	Raw264.7<br>40% FBS	Raw264.7<br>fMLP	PM	PM<br>IL4	PM<br>IL13	PM<br>LPS	PM<br>P	PM<br>A	PM<br>A+P
PM<br>40% FBS	PM<br>fMLP	THP1	THP1<br>IL4	THP1<br>IL13	THP1<br>LPS	THP1<br>P	THP1<br>A	THP1<br>A+P	THP1<br>40% FBS
THP1<br>fMLP	LSEC	LSEC<br>P	LSEC<br>A	LSEC<br>A+P	C166	C166<br>P	C166<br>A	C166<br>A+P	ESC-MSC<br>A+P

Table S1. Information of cell candidates screened by CEDSS. Cell candidates of CEDSS assay. Detailed settings of experiments could be referred to experimental section (priming cells). Cell types: human promyelocytic cell line (HL60), mouse macrophage cell line (Raw264.7), human hepatic stellate cell line (LX-2), human liver sinusoidal endothelial cell line (LSEC), hESC-derived MSCs (ESC-MSC), hESC-derived LSECs (ESC-LSEC), mouse yolk sac-derived endothelial cell (C166), mouse primary macrophages (PM), human adipose-derived mesenchymal stem cells (ADMSC), human monocytic cell line (THP-1). Cell priming factors: 200× Accutase (A), 50 ng/ml PMA (P), 2 μM fMLP, 20 ng/ml LPS, 20 ng/ml IL4, 20 ng/ml IL13, 20 ng/ml TGFβ, 1.3 % DMSO, 40% FBS.

Table S2. Primer set for gene expression analysis using Real-Time qPCR

	Gene	Forward	Reverse
Human genes	*c-Fos*	CCGGGGATAGCCTCTCTTACT	CCAGGTCCGTGCAGAAGTC
	ACTA2	AAAAGACAGCTACGTGGGTGA	GCCATGTTCTATCGGGTACTTC
	COL1A1	GAGGGCCAAGACGAAGACATC	CAGATCACGTCATCGCACAAC
	COL3A1	GGAGCTGGCTACTTCTCGC	GGGAACATCCTCCTTCAACAG
	MMP1	AAAATTACACGCCAGATTTGCC	GGTGTGACATTACTCCAGAGTTG
	MMP2	TACAGGATCATTGGCTACACACC	GGTCACATCGCTCCAGACT
	MMP3	AGTCTTCCAATCCTACTGTTGCT	TCCCCGTCACCTCCAATCC
	MMP9	TGTACCGCTATGGTTACACTCG	GGCAGGGACAGTTGCTTCT
	MMP10	TGCTCTGCCTATCCTCTGAGT	TCACATCCTTTTCGAGGTTGTAG
	MMP13	ACTGAGAGGCTCCGAGAAATG	GAACCCCGCATCTTGGCTT
	MMP14	GGATACCCAATGCCCATTGGCCA	CCATTGGGCATCCAGAAGAGAGC
	TIMP1	CTTCTGCAATTCCGACCTCGT	ACGCTGGTATAAGGTGGTCTG
	TIMP2	AAGCGGTCAGTGAGAAGGAAG	GGGGCCGTGTAGATAAACTCTAT
	TIMP4	CCACTCGGCACTTGTGATTC	CATCCTTGACTTTCTCAAACCCT
	IL6	ACTCACCTCTTCAGAACGAATTG	CCATCTTTGGAAGGTTCAGGTTG
	CSF2	TCCTGAACCTGAGTAGAGACAC	TGCTGCTTGTAGTGGCTGG
	CXCL2	CGCCCATGGTTAAGAAAATCA	CCTTCTGGTCAGTTGGATTTGC
	GAPDH	GGCTGAGAACGGGAAGCTTGTCAT	CAGCCTTCTCCATGGTGGTGAAGA
Mouse genes	*Acta2*	GTCCCAGACATCAGGGAGTAA	TCGGATACTTCAGCGTCAGGA
	Col1a1	GCTCCTCTTAGGGGCCACT	CCACGTCTCACCATTGGGG
	Col3a1	CTGTAACATGGAAACTGGGGAAA	CCATAGCTGAACTGAAAACCACC
	Gapdh	TCACCACCATGGAGAAGGC	GCTAAGCAGTTGGTGGTGCA
	Il1b	GCAACTGTTCCTGAACTCAACT	ATCTTTTGGGGTCCGTCAACT
	Alb	TGCTTTTTCCAGGGGTGTGTT	TTACTTCCTGCACTAATTTGGCA
	Cyp27a1	CCAGGCACAGGAGAGTACG	GGGCAAGTGCAGCACATAG
	Hgf	ATGTGGGGGACCAAACTTCTG	GGATGGCGACATGAAGCAG
	TNFa	CCCTCACACTCAGATCATCTTCT	GCTACGACGTGGGCTACAG
	Il6	TAGTCCTTCCTACCCCAATTTCC	TTGGTCCTTAGCCACTCCTTC
	Arg1	CTCCAAGCCAAAGTCCTTAGAG	AGGAGCTGTCATTAGGGACATC
	Il10	GCTCTTACTGACTGGCATGAG	CGCAGCTCTAGGAGCATGTG
	Mrc1	CTCTGTTCAGCTATTGGACGC	CGGAATTTCTGGGATTCAGCTTC
	Ccl2	TTAAAAACCTGGATCGGAACCAA	GCATTAGCTTCAGATTTACGGGT
	Ccl5	GCTGCTTTGCCTACCTCTCC	TCGAGTGACAAACACGACTGC

Movie S1. Dynamic degradation of collagen matrix mediated by nLSECs. nLSECs (green). Collagen I (red). Scale bars, 500 μm.

Movie S2. Dynamic degradation of collagen matrix mediated by dLSECs. dLSECs (green). Collagen I (red). Scale bars, 500 μm.
